# Structure-Property-Function Relationship in Humic Substances to Explain the Biological Activity in Plants

**DOI:** 10.1038/srep20798

**Published:** 2016-02-10

**Authors:** Andrés Calderín García, Luiz Gilberto Ambrosio de Souza, Marcos Gervasio Pereira, Rosane Nora Castro, José María García-Mina, Everaldo Zonta, Francy Junior Gonçalves Lisboa, Ricardo Luis Louro Berbara

**Affiliations:** 1 Federal Rural University of Rio de Janeiro, Soil Science Dept. Rodovia BR 465 km 7, Seropédica, RJ, CEP 23890-000, Brazil; 2 Federal Rural University of Rio de Janeiro, Chemistry Department, Rodovia BR 465 km 7, Seropédica, RJ, CEP 23890-000, Brazil; 3 Department of Environmental Biology, Agricultural Chemistry and Biology Group-CMI Roullier, Faculty of Sciences, University of Navarra, Pamplona, Navarra 31008, Spain.

## Abstract

Knowledge of the structure-property-function relationship of humic substances (HSs) is key for understanding their role in soil. Despite progress, studies on this topic are still under discussion. We analyzed 37 humic fractions with respect to their isotopic composition, structural characteristics, and properties responsible for stimulating plant root parameters. We showed that regardless of the source of origin of the carbon (C_3_ or C_4_), soil-extracted HSs and humic acids (HAs) are structurally similar to each other. The more labile and functionalized HS fraction is responsible for root emission, whereas the more recalcitrant and less functionalized HA fraction is related to root growth. Labile structures promote root stimulation at lower concentrations, while recalcitrant structures require higher concentrations to promote a similar stimulus. These findings show that lability and recalcitrance, which are derived properties of humic fractions, are related to the type and intensity of their bioactivity. In summary, the comparison of humic fractions allowed a better understanding of the relationship between the source of origin of plant carbon and the structure, properties, and type and intensity of the bioactivity of HSs in plants. In this study, scientific concepts are unified and the basis for the agronomic use of HSs is established.

The functions of dissolved organic matter (DOM) in soils, specifically humic substances (DOM-HSs), are well established^1^,^2^,^3^,^4^. Several studies show that DOM-HSs regulate metabolic processes related to plant growth, especially the emission and growth of the root system^5^,^6^,^7^,^8^,^9^,^10^,^11^,^12^. The capacity of HSs to trigger stimuli in plant metabolism is directly related to their structure. HSs extracted from soils and with structural predominance of –CH_3_ and –COOH carbons stimulate carbon metabolism in *Pinus nigra* plants^13^, whereas the carbons belonging to lignin structures and –COOH groups in vermicompost humic acids (HAs) positively correlate with the emission of lateral roots in maize plants^14^.

The properties related to the structure of HSs are also related to their bioactivity. HAs isolated from composted materials structurally enriched in carboxyl (–COOH) groups and hydrophobic structures stimulated root growth in maize plants^15^, whereas hydrophobic structures in HSs extracted from vermicompost are responsible for stimulating the proton pumps in roots^16^. Zancani *et al*.^17^ showed that embryogenic cell multiplication results from the hydrophilicity and labile conformations in soil-extracted fulvic acids, and García *et al*.^18^,^19^ showed that aliphatic and oxygenated structures in vermicompost HAs are related to the protective effects on rice plants subjected to water stress.

Studies on the structure-property-function relationship of HSs in plants are of great importance in understanding their modes of action and practical use. This study aimed to investigate the relationship between the structure of humic fractions from soils and composted materials and the properties regulating and defining the bioactivity at the root level in rice plants.

A total of 37 humic fractions (HS, HA and Humins-Hus) derived from Histosols from different sources and composted materials were characterized in this study using isotopic, chemical and spectroscopic methods (elemental analysis, ultraviolet–visible [UV-vis] spectroscopy, Fourier transform infrared spectroscopy [FTIR] and carbon-13 cross polarization – magic angle spinning nuclear magnetic resonance [^13^C-CP/MAS-NMR]). Chemometric methods were used to relate the properties with the different root parameters in plants. We also discuss the relationship between the type and intensity of plant bioactivity and the plant carbon origin, structural characteristics and recalcitrance and lability properties.

## Results

### Structural differences between humic fractions from different sources observed by ^13^C-NMR spectroscopy

Soil humic fractions showed the following ranking of aromaticity: HA > HS > Hu. The spectral signatures of humic fractions correspond to the presence of sp^3^ and sp^2^ carbon (see spectra included in the Supplementary material – SM, Fig. S1). The structural characteristics of soil humic fractions showed that HAs have a higher predominance of unsubstituted C-aliphatic and C-aromatic groups than HS and Hu fractions. HAs predominantly had unsubstituted C-aromatic groups and the most striking aromatic properties among the humic fractions extracted from composted materials (HAs and HSs).

**Figure 1:**
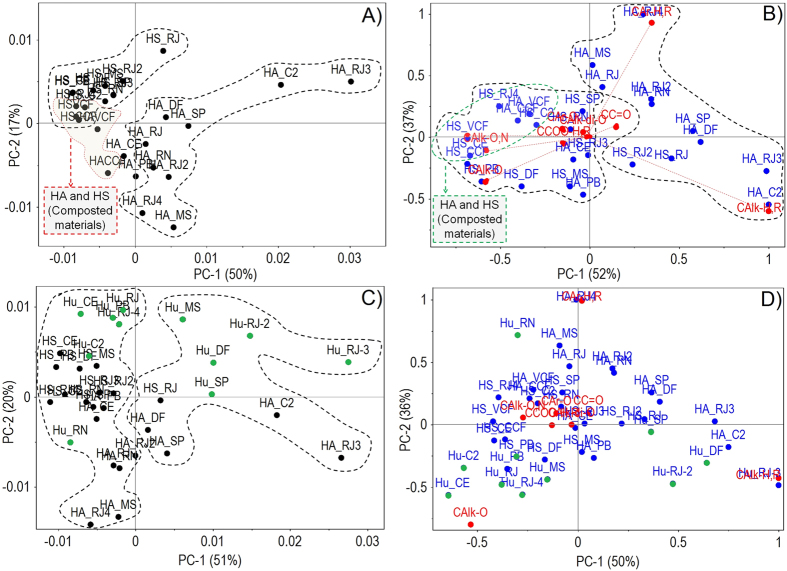
PCA for the data obtained by loading the ^13^C-CP/MAS-NMR spectra of HSs from Histosols and composted materials. (**A,C**) PCA performed using pure spectra. (**B,D**) PCA performed through integration of regions of pure spectra.

The structural characteristics of soil-extracted humic fractions and those extracted from composted materials indicated that unsubstituted C-aliphatic and C-aromatic groups predominate in soil HAs. However, vermicompost HAs had aromatic characteristics that were more striking than those extracted from soils. An average predominance of unsubstituted C-aliphatic groups stood out among the soil HS fractions (see Supplementary material Table S1). ^13^C-CP/MAS-NMR spectral data confirmed this observation upon multivariate analysis (Fig. 1, see Supplementary material Fig. S2). The principal component analysis (PCA) plot (73% of the total variance explained) (see Supplementary material Fig. S1A) showed a clustering of ten of the thirteen studied HAs with negative values in the PC1 (57%), wherein HAs extracted from composted materials were included. Fig. S1-A1 (see Supplementary material) shows a PCA with 92% of the total variance explained based on the relative number of types of carbons of each HA. Six soil HAs were clustered in PC1 (60%) because of the predominance of C-alkyl-O and C-alkyl structures, whereas another five HAs were clustered with negative values because of the predominance of C-aromatic groups. HAs extracted from composted materials were more related to substituted C-aromatic and C-aliphatic groups.

HS fractions were distributed into two clusters in the PCA (76% of the total variance explained) of pure spectra (see Supplementary material Fig. S1B). In contrast to the behavior of HA fractions, six HS fractions were more related to unsubstituted C-aromatic and C-aliphatic groups in PC1 (72%). The remaining HS fractions, including those extracted from composted materials, were closely related to substituted C-aromatic and C-aliphatic groups (see Supplementary material Fig. S1-B1).

The Hu fraction showed a distribution in the PCA with 86% of the total variance, with PC1 (72%) similar to that shown by HAs (see Supplementary material Fig. S1C). Five Hu fractions were clustered and related to C-aliphatic groups (positive values), while the remaining fractions were clustered and related (negative values) to C-aromatic and C-aliphatic groups (PC1 72%).

Figure 1A shows the PCA (67% of the total variance explained) of pure spectra for the soluble fractions HA and HS. HA fractions were clustered in positive values, whereas HS fractions were clustered with negative values in PC1 (50%). HAs extracted from vermicompost and compost showed a closer relationship with the soil-extracted HS fractions. In the PCA in Fig. 1B, PC1 (52%) showed that soil HAs were related to C-aliphatic and unsubstituted C-aromatic groups, while soil HSs, HSs and HA from composted materials were related to the more functionalized C-aliphatic and C-aromatic groups. The PCA (71% of the total variance explained) in Fig. 1C shows the three fractions studied. Five Hu fractions and three HA fractions were clustered with positive values in PC1 (51%), while the three fractions (HS, HA and Hu) were related to negative values. The PCA summarized in Fig. 1D (86% of the total variance explained) shows a cluster in PC1 (50%), with positive values of Hus and HAs with the same origin and closely related to unsubstituted C-aliphatic groups. Another Hu group was clustered with the HSs, with positive values in PC1 and closely related to C-functionalized groups.

### Lability and recalcitrance of humic fractions analyzed by ^13^C-NMR spectroscopy combined with MCR

**Figure 2:**
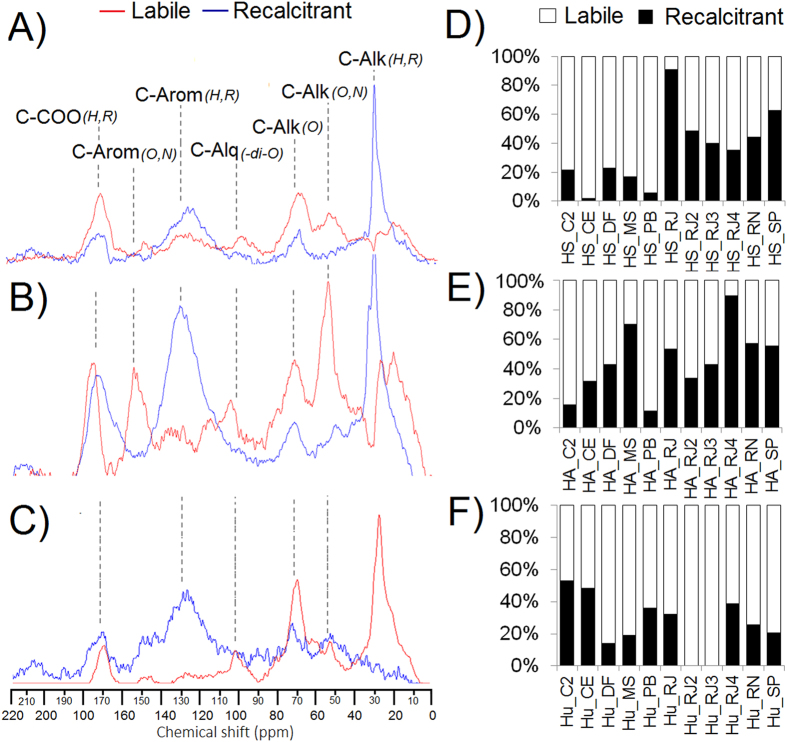
Multivariate curve resolution (MCR) performed by loading the ^13^C-CP/MAS-NMR spectra of HSs from Histosols and composted materials. (**A**) MCR of HSs, (**B**) MCR of HAs and (**C**) MCR of Hus.

HSs showed recalcitrance resulting from the unsubstituted C-aromatic and C-aliphatic groups and lability primarily resulting from the substituted C-aliphatic (C-alkyl O, N and C-alkyl-O) groups and C of carboxyl groups (Fig. 2A). Conversely, the recalcitrance of HAs not only resulted from the unsubstituted C-aromatic and C-aliphatic groups but also showed the contribution of C from carboxyl groups, while lability resulted from the unsubstituted C-aliphatic and C-aromatic groups (Fig. 2B). The patterns of recalcitrance and lability of Hus were significantly less evident because the largest contribution to recalcitrance resulted from both C-aromatic groups and substituted aliphatic structures and carboxylic C. In turn, the largest contribution to lability resulted from substituted and unsubstituted C-aliphatic groups and from carboxylic C (Fig. 2C).

The quantifications of lability and recalcitrance (%) corroborated the differences observed in the MCR of humic fractions (Fig. 2D,E,F). Soil-extracted HSs showed ~56% lability and ~24% recalcitrance, while HAs showed ~47% lability and ~39% recalcitrance, and Hus showed 67% lability and 32% recalcitrance. The fractions showed the following ranking of recalcitrance: HA > Hu > HS.

### Structural differences between humic fractions from different sources observed with FTIR spectroscopy

The spectral characteristics of humic fractions showed the presence of functional groups of different chemical natures (see spectra included in Supplementary material, Fig. S2). Figure S4 (see Supplementary material) shows the PCA (89% of the total variance explained) for the HA fraction. Nine HAs were clustered with positive values in PC1 (79%) and four with negative values. Unlike PCAs performed using the ^13^C-NMR spectra, the HAs derived from composted materials showed no similarities in terms of functional groups in this analysis (see Supplementary material Fig. S4-A). The PCA (75% of the total variance explained) for the HS fractions showed a clear separation of these fractions into two groups: seven HS fractions clustering with positive values in PC1 (56%) and six with negative values. The HSs derived from composted materials also showed no similarities in terms of functional groups (see Supplementary material Fig. S4-B). The PCA (86% of the total variance explained) also showed that Hus were distributed into two clusters in PC1 (65%). Six Hus were clustered with positive values and five with negative values (see Supplementary material Fig. S4-C).

**Figure 3:**
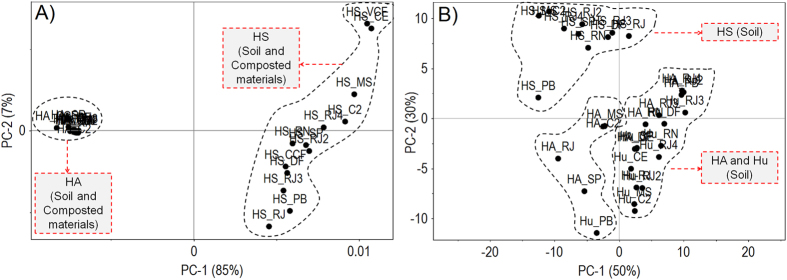
PCA of the data obtained by loading the FTIR spectra of HSs from Histosols and composted materials. (**A**) soluble fractions, HAs and HS. (**B**) three fractions, HAs, HS and Hu.

The comparison between the HA and HS fractions in the PCA (92% of the total variance explained) showed a clear separation of these fractions in PC1 (85%) (Fig. 3A). HSs and HAs showed strong differences in terms of functional groups. HSs clustered with positive values in PC1, while HAs clustered with negative values. The PCA of the three fractions (80% of the total variance explained) showed that Hus were similar to HAs in terms of functional groups, clustering in close relationship in PC1 (50%).

### Structural differences between humic fractions from different sources observed upon isotopic and elemental analysis

The δ^13^C isotopic compositions were similar in the three soil-extracted humic fractions (see Supplementary material Table S2). In general, these humic fractions had isotopic compositions between −20% and −30%, while the fractions isolated from composted materials had compositions between −14% and −16%. This isotopic composition showed that the plant carbon of soil humic fractions was likely derived from C_3_ photosynthetic pathway plants, while the carbon of humic fractions from composted materials was derived from C_4_ plants^20^ (see Supplementary material Table S2).

The HSs fractions extracted from composted materials had higher C values than those extracted from Histosols, whereas HS_VCF had higher quantities of N. The H/C ratio was lower in the HSs extracted from composted materials, ν was slightly higher in HS_CCF, and δ was slightly higher in HS_VCF than in soil-extracted HSs. The fractions HS_CCF and HS_VCF had lower E_4_/E_6_ ratios.

HAs extracted from composted materials had higher levels of C and N than those in soil-extracted HAs. The HA_CCF fraction had the highest levels of O, a higher O/C ratio and a higher ω value, while HA_VCF showed the highest values of δ and E_4_/E_6_ ratio.

**Figure 4:**
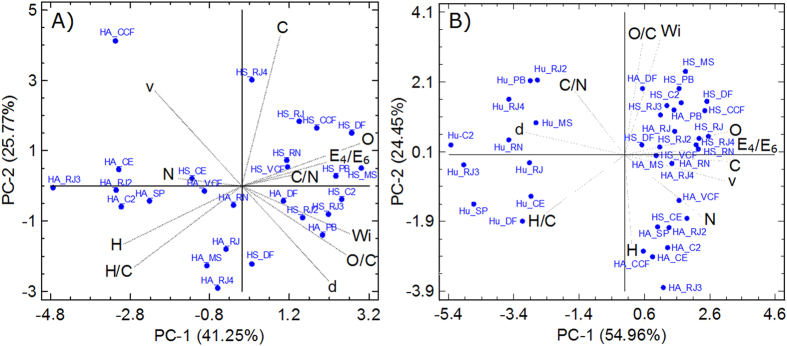
PCA of the data derived from the elemental composition of HSs from Histosols and composted materials. (**A**) soluble fractions, HAs and HS. (**B**) three fractions, HAs, HS and Hu.

Figure 4 shows how the elements were related to each soluble humic fraction (Fig. 4A) and between the three humic fractions (Fig. 4B). The PCA (67.02% of the total variance explained) performed using the HS and HA fractions indicated the existence of a relationship between the HSs and the parameters associated with oxygenation/functionalization (O, O/C and ω), with positive values of PC1 (41.25%) and a relationship with the parameters C/N and E_4_/E_6_ ratios, C and δ. In turn, the HAs showed a relationship with the parameters related to bond saturation (H, H/C), ν and N content.

The PCA (79.41% of the total variance explained) performed using the three fractions (Fig. 4B) showed that the soluble HS and HA fractions were clustered with positive values in PC1 (54.96%) with all elements present (C, H, N, O) and with the ω, ν and E_4_/E_6_ parameters. Hus clustered independently with negative values in PC1, showing a relationship with the parameters C/N, H/C and δ.

### Bioactivity of HS and HA humic fractions in the root system of rice plants and its relationship with structural characteristics

The effects exerted by the HS and HA humic fractions on the root system of rice plants are shown in Fig. S3 (see Supplementary material). The most promising concentrations of HSs stimulated the root parameters in the range of 1.5–5.0 mg (C). L^−1^, while the HAs exerted stimulus at higher concentrations of 5.0 and 10.0 mg (C). L^−1^.

**Figure 5:**
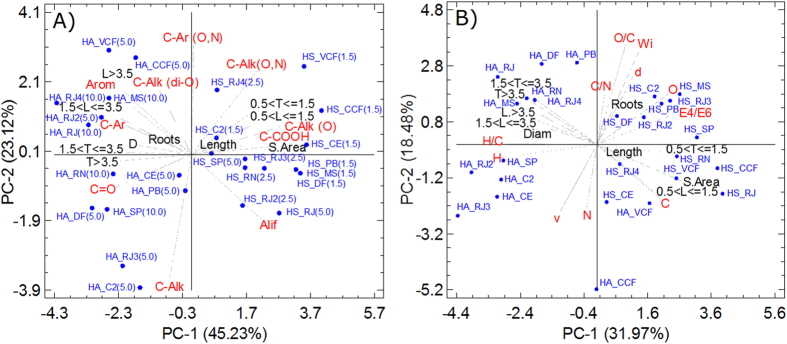
PCA showing the relationship between the data resulting from the quantification of carbon types based on the ^13^C-CP/MAS-NMR spectra (**A**) and the elemental analysis (**B**) of humic fractions (HSs and HAs), and the root parameters evaluated in rice plants.

Figure. 5A,B show the PCAs relating the humic fractions with the root parameters, the types of carbon in ^13^C-CP/MAS-NMR, and the elemental composition. The PCA in Fig. 5A (68.35% of the total variance explained) shows a clustering of HS fractions with positive values and HA fractions with negative values in PC1. The fraction of HSs extracted from soils and composted materials showed a close relationship (stimulus) with the root parameters corresponding to surface area (S.Area), radicle length (Length) and smaller roots (0.5 < T < 1.5, 0.5 < L < 1.5, T and L are the numbers of roots and the roots with lengths between 0.5 and 1.5, respectively). These stimuli were also closely related to substituted C-aliphatic (C-Alk [O,N], C-Alk-O) and carboxylic C-COOH groups and the aliphaticity. The HA fraction extracted from soils and composted materials showed a relationship with root parameters diameter (D), root number (roots) and larger roots (1.5 < T < 3.5, 1.5 < L < 3.5, L > 3.5, T > 3.5). These stimuli were closely related to the substituted C-aliphatic (C-Alk [di-O]), unsubstituted C-aliphatic (C-Alk), C-aromatic and carbonyl CC = O groups and to aromaticity.

Figure 5B shows the PCA (50.45% of the total variance explained) for the elemental composition data and root bioactivity parameters. The HS and HA fractions extracted from composted materials were clustered with negative values, and soil-extracted HAs were clustered with negative values in PC1 (31.97%). The HS and HA fractions extracted from composted materials showed close relationships (stimuli) with root surface area, radicle length, root number and roots of smaller size and diameter (0.5 < T < 1.5, 0.5 < L < 1.5); in turn, these parameters were related to C and O levels, C/N and O/C ratios, E_4_/E_6_ properties and apparent density (d). Soil-extracted HAs were related to the number of larger roots and root diameter (Diam); in turn, these parameters were related to H and C levels, the H/C ratio and apparent volume (v).

### Structure-property-function relationship of HSs and HAs

**Figure 6:**
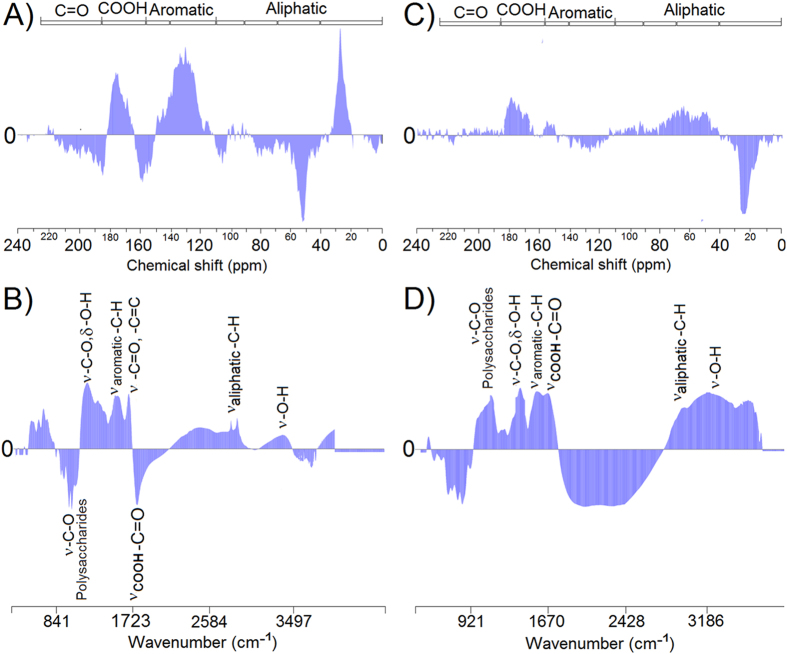
Principal component regression (PCR) of data from the ^13^C-CP/MAS-NMR and FTIR spectra and the root parameters of plant bioactivity. (**A, B**) Humic acids (HAs) and (**C,D**) Humic substances (HS).

Figure 6 shows the principal component regression (PCR) between the spectral data and the biological activity parameters in the root system. The PCR performed between ^13^C-NMR and the root parameters regarding the HA fractions (Fig. 6A) revealed that the carbon types that positively correlated with the root parameters were unsubstituted C-aliphatic, unsubstituted C-aromatic and carboxylic C. In contrast, the PCR for the HS fractions (Fig. 6C) showed that the carbon types that positively correlated with the root parameters were substituted C-aliphatic, substituted C-aromatic and carboxylic C.

Figure 6B shows the PCR performed using the FTIR data of HAs and root parameters. The functional groups positively correlated with the root parameters were the stretching vibrations –OH, –CH, C = O, C = C, aromatic CH and C–O. The PCR performed using the HS data revealed that most functional groups in the spectral region were positively correlated with the root parameters (the stretching vibrations –OH, –CH, C = O, C = C, aromatic CH and C–O alcohols and polysaccharides) (Fig. 6D).

## Discussion

The type of plant material that originated the humic fractions had no effect on the type of structure. Soil HSs derived from C_3_ plant carbon develop a structure similar to the HSs from composted materials derived from C_4_ plant carbon, and the same trend is observed for the HA fraction.

The HA fraction extracted from Histosols and composted materials is predominantly aromatic and aliphatic, with low chemical functionalization (substitution by O and N), while the HS fraction predominantly consists of functionalized structures (see Supplementary material Fig. 1B and Table S2). These structural characteristics indicate that the HSs fraction is more labile than the HA fraction and that the HA fraction has essentially recalcitrant compounds (Fig. 2). The Hu fraction showed no specific structural characteristics differentiating it from the HA fraction. The PCA-FTIR showed a close relationship between this fraction and the HA fraction, indicating that its lability and recalcitrance properties were less evident. These results confirm previous studies on the relationship between Hus and HAs^21^,^22^,^23^.

HSs extracted from both soils and composted materials showed similarities in their structural characteristics. This finding indicates that HSs produce similar humic structures, regardless of their source of origin, which was not observed for HAs. HAs extracted from composted materials showed greater structural similarity with the HS fractions than with soil-extracted HA fractions. These results indicate that the greatest structural changes occur when performing chemical fractionation using the HSs fraction.

In summary, the structural differences between the HSs fraction and the HA fractions (soluble fractions) are not related to the presence of structures (see Supplementary material Fig. S1) but rather to their conformation and/or structural organization. These results reinforce the structural interpretation of humic fractions as supramolecules, as reported by Nebiosso & Piccolo^24^,^25^. The classical interpretation of the Hu fraction as an independent fraction was less evident. Conversely, the Hus had lower aromaticity and structural complexity, as reported in the studies by Hayes *et al*.^23^ and Nebbioso *et al*.^22^. The results obtained in this study suggest that the Hu fraction is similar to the HA fraction but that it already formed bonds with the soil mineral fraction, which would explain its low solubility^22^.

For the first time, this study shows that the recalcitrance and lability of humic fractions are chemical properties that define the stimulation of plant root parameters. The root length and emission of smaller roots are related to less complex and functionalized structures (-O, -N functionalized aliphatic chains) (Fig. 5A) and higher E_4_/E_6_ ratios (Fig. 5B), and these structures are responsible for structural lability. Conversely, the growth of larger roots is related to more complex structures and lower chemical functionalization (unsubstituted aromatic and aliphatic groups) (Fig. 5A). The PCR analysis confirmed this relationship because the spectral pattern recorded for the structures that was positively correlated with the root stimulus in the HSs corresponds to the lability pattern recorded in these substances. Thus, the spectral pattern recorded for the structures that was positively correlated with the root stimulus in the HAs is related to the recalcitrance pattern recorded for this fraction (Fig. 6).

The relationship between recalcitrance and lability also defined the maximum level of bioactivity in each humic fraction. The HA fraction stimulated the plant root parameters at concentrations (5.0–10.0 mg (C) L^−1^) five times higher than the stimulus concentrations for HSs (1.5–2.5 mg (C) L^−1^). This relationship between recalcitrance and lability was even more evident when analyzing the same fraction. The most labile HS fractions (HS_RJ, HS_SP, HS_RN, HS_RJ4, HS_RJ3, HS_RJ2) promoted root stimulation at lower concentrations (1.5 mg [C] L^−1^), while the most recalcitrant fractions required higher concentrations to promote a similar stimulus. The same trend was observed in the HA fractions.

The results of this study on the action of HSs on plant root growth and development are explained in other studies that show these effects. For example, auxin-type effects (hormonal effects) that are well established and proven in the literature can explain this type of action of HSs on the root system^26^,^27^, as well as the nutritional effects shown by the HSs on NO_3_^−^ uptake and Fe metabolism^28^,^29^.

Lastly, this study demonstrates that the plant material source (C_3_ or C_4_) had no clear effect on the structural characteristics of humic fractions, which are similar to each other. However, the properties generated from these structures are different. Thus, one can conclude that their main differences lie in their structural organization, which may be understood as supramolecularity. The supramolecularity of humic fractions, which involves the interaction between molecules and spatial reorganization^24^,^25^, is a structural characteristic of humic fractions that is able to define properties such as recalcitrance and lability. Simultaneously, these properties define the type and magnitude of plant bioactivity. Thus, the structure-property-function relationship of the humic fractions studied is established and proven.

## Materials and Methods

### Soil and composted materials used to prepare the humic fractions

The soils selected to prepare the humic fractions are classified as Histosols^30^ (see the characteristics of the soils in the Supplementary material—Table S3). The soil samples were collected in the histic horizons (0.00–0.40 m) from seven Brazilian states (Rio de Janeiro, Brasília-DF (Federal District), Mato Grosso do Sul, São Paulo, Paraíba, Rio Grande do Norte, Ceará) with variations in temperature, humidity and rainfall^31^,^32^. The humic fractions were prepared from composted materials resulting from a vermicompost produced using cattle manure and elephant grass (*Pennisetum purpureum*) as raw materials (see the characteristics of the materials in the Supplementary material—Table S4).

The nomenclature used for the different humic fractions was as follows. Humic substances: (Rio de Janeiro: HS_RJ, HS_RJ2, HS_RJ3 and HS_RJ4; Brasília-DF: HS_DF; Mato Grosso do Sul: HS_MS; São Paulo: HS_SP; Paraíba: HS_PB; Rio Grande do Norte: HS_RN; Ceará: HS_CE, HS_C2; Vermicompost: HS_VCF; Compost: HS_CCF). Humic acids: (Rio de Janeiro: HA_RJ, HA_RJ2, HA_RJ3 and HA_RJ4; Brasília-DF: HA_DF; Mato Grosso do Sul: HA_MS; São Paulo: HA_SP; Paraíba: HA_PB; Rio Grande do Norte: HA_RN; Ceará: HA_CE, HA_C2; Vermicompost: HA_VCF; Compost: HA_CCF). Humins: (Rio de Janeiro: Hu_RJ, Hu_RJ2, Hu_RJ3 and Hu_RJ4; Brasília-DF: Hu_DF; Mato Grosso do Sul: Hu_MS; São Paulo: Hu_SP; Paraíba: Hu_PB; and Rio Grande do Norte: Hu_RN; Ceará: Hu_CE, Hu_C2).

### Preparation and purification of humic fractions

The extraction and purification of humic fractions – humic substances (HSs) and humic acids (HAs) – were performed following the method of the International Humic Substances Society (IHSS) and according to the protocol reported by Swift^33^. An initial modification was adopted and consisted of pretreating the soil samples using an HCl solution (0.1 mol L^−1^), pH 1.0–2.0^34^,^35^. The humins (Hu) were obtained and purified using the procedure described by Nebiosso *et al*.^36^ with modifications. These procedures are described in detail in the Supplementary materials.

### Quantification of stable ^13^C isotopes (δ^13^C)

The ^13^C (δ^13^C) isotopic abundance was assessed in the samples of humic fractions extracted from soils and composted materials using 200- to 400-μg samples and an automated Carlo Erba C–N analyzer (EA 1108, Milan, Italy) coupled to a continuous-flow isotope ratio-mass spectrometer (Finnigan Mat, Bremen, Germany). The results are expressed as δ^13^C (%) using Pee Dee Belemnite (PDB) as a reference standard for carbon.

### Elemental composition and E_4_/E_6_ ratio

The elemental analysis was obtained using a Perkin Elmer 2400 CHN elemental analyzer. The analyses were performed using 1.1 ± 0.1 mg of samples weighed in a micro-balance coupled to the device. The reference standard used was acetanilide (C: 71.09%; H: 6.71%; N: 10.36%). The degree of internal oxidation (Wi) and density of the humic fraction were determined according to Orlov^37^. The analysis of HSs and HAs was performed using Ultraviolet-visible (UV-vis) spectroscopy, and the spectra were recorded according to Canellas^38^. The UV-vis spectra were recorded in a spectral range of 200 to 800 nm. The absorbance at 465 nm was divided by the value measured at 665 nm to determine the E_4_/E_6_ ratio coefficient.

### Fourier transform infrared spectroscopy (FTIR) and carbon-13 cross-polarization—magic angle spinning nuclear magnetic resonance (^13^C-CP/MAS-NMR) spectroscopy

The spectra in the infrared region were recorded in the range of 4.000–400 cm^−1^ in an NICOLET infrared spectrometer (FT-IR), model 6700, with Fourier transform (FTIR), using KBr pellets (5 mg of freeze-dried HA + 200 mg de KBr).

^13^C-CP/MAS-NMR spectroscopy was performed using a Bruker AVANCE II NMR device at 400 MHz, equipped with a 4-mm Narrow MAS probe and operating in ^13^C magnetic resonance sequence at 100.163 MHz. Spectra were divided into chemical shifts; the areas were determined after integration of each region and are expressed as percentages of total area. The regions were assigned as follows: alkyl C (C_Alq-H,R_): 0–45 ppm; methoxyl and N–alkyl C (C_Alq-O,N_): 45–60 ppm; O–alkyl C (C_Alq-O_): 60–91 ppm; di–O–alkyl C (anomeric) (C_Alq-di-O_): 91–110 ppm; aromatic C (C_Ar-H,R_): 110–142 ppm; O–aromatic C (C_Ar-O_): 142–156 ppm; carboxyl C (C_COO-H,R_): 156–186 ppm and carbonyl C (C_C_ = _O_): 186–230 ppm.

### Biological activity experiments of soluble humic fractions in rice plants

The experiments of bioactivity of HSs and HAs in rice plants (*Oryza sativa* L.) were conducted using the Piauí rice variety. The plants were grown in a growth chamber with the following conditions: light cycle: 12/12 h (light/dark), photosynthetic photon flux: 250 μmol m^−2 ^s^−1^, relative humidity: 70%, and temperature: 28 °C/24 °C (day/night). Rice seeds were previously disinfected with sodium hypochlorite (2%) for 10 minutes and subsequently washed with distilled water. Four days after seed germination, the seedlings were treated using a Hoagland^39^ solution modified to ¼ of the total ionic strength (pH = 5.5). Three days later, the Hoagland solution was replaced with a solution with ½ of the total ionic strength, and this solution was replenished throughout the experiment. The experimental design used in all experiments was a completely randomized design, using a total of five plants per pot and five replicates per treatment.

Preliminary experiments were conducted for testing a range of concentrations (0.5, 1.5, 2.5, 5.0 and 10.0 mg [C-HA/HS] L^−1^) (see Supplementary material Fig. S6) to determine the concentrations with the most promising responses in stimulating the root systems of rice plants. The rice plants were placed in contact with the nutrient solutions containing the dissolved HAs and HSs. The plants were removed to assess the root parameters after completing ten days of growth following transplantation (days after transplantation-DAT). New experiments were conducted to evaluate the response of the root systems of rice plants to soluble humic fractions based on the results from the previous experiment, wherein the HA and HS concentrations that promoted the highest root number were applied.

### Assessment of the root parameters of rice plants

An Epson Expression 10000XL scanning system with an additional light unit (Turbo Pascal Unit, TPU) was used. Four different root traits were analyzed and quantified: length (mm), surface area (mm^2^), mean diameter (mm), and root number. Length (mm) and root number were defined and measured using the classes superfine (0.5–1.5 mm), fine (1.5–3.5 mm) and coarse (>3.5 mm) with the software WinRhizo Arabidopsis, 2012b (Régent Instruments, Inc., Quebec, Canada).

### Chemometric treatments of spectral data and biological activity

Principal component analyses (PCA), multivariate curve resolution (MCR) and principal component regression (PCR) using the ^13^C-CP/MAS-NMR and FTIR spectral data of the humic fractions were performed using the software Unscrambler^®^ X 10.3 package (Camo Software AS Inc., Oslo, Norway). The ^13^C-NMR and FTIR spectra of humic fractions were loaded using the software and were area-normalized. The range selected to conform the ^13^C-NMR spectral data matrix was from −20 ppm to 240 ppm. The values outside of this range were discarded to avoid false contributions to the analyses. The PCA of each humic fraction was performed using a non-linear iterative partial least squares (NIPALS) algorithm and the CROSS VALIDATION method with a maximum of seven components. The range from 400 cm^−1^ to 3800 cm^−1^ was selected for the FTIR PCA. The conditions of analysis were the same as those used for ^13^C-NMR. The lability and recalcitrance (%) were quantified based on the concentration of the components. The PCR analyses were performed with the FTIR and ^13^C-NMR data using the normalized bioactivity parameters evaluated as predictor variable X according to Khattree and Naik^35^. The spectral data were used as predictor variable Y. A maximum of seven principal components were used for a 95% confidence level. The NIPALS algorithm and leverage correction validation were used.

Multivariate analyses without involving spectral data loading (PCA of the elemental composition of humic fractions and PCA of the root parameters evaluated in the bioactivity experiments) were performed using the Statgraphics® Centurion XVI package (StatPoint Technologies, Inc. 560 Broadview Ave # 201, Warrenton, VA 20186, USA). The data were loaded into Statgraphics® and the analyses were performed with the homogenized data using their standard deviations^40^. The parameters evaluated were selected in both analyses, and the samples of humic fractions were selected as points levels. A maximum of ten components and a bi-plot chart type were selected for graphical representation.

## Additional Information

**How to cite this article**: García, A. C. *et al*. Structure-Property-Function Relationship in Humic Substances to Explain the Biological Activity in Plants. *Sci. Rep.* **6**, 20798; doi: 10.1038/srep20798 (2016).

## Supplementary Material

Supplementary Information
