# Supplementary material for: Structure-Property-Function Relationship in Humic Substances to Explain the Biological Activity in Plants
Source: Sci Rep. 2016 Feb 10;6:20798. doi: 10.1038/srep20798 (PMC4748406; doi:10.1038/srep20798)
Supplement: Supplementary Information [file srep20798-s1.pdf]

Supplementary Information

Structure-Property-Function Relationship in Humic Substances to Explain the Biological Activity in Plants

Andrés Calderín García[1*], Luiz Gilberto Ambrósio de Souza[1], Marcos Gervasio Pereira[1], Rosane Nora Castro[2], José María García-Mina[3], Everaldo Zonta[1], Francy Junior Gonçalves Lisboa[1], Ricardo Luis Louro Berbara[1]

[1] Federal Rural University of Rio de Janeiro, Soil Science Dept. Rodovia BR 465 km 7, Seropédica, RJ, CEP 23890-000, Brazil.

[2] Federal Rural University of Rio de Janeiro, Chemistry Department. Rodovia BR 465 km 7, Seropédica, RJ, CEP 23890-000, Brazil.

[3] Department of Environmental Biology. Agricultural Chemistry and Biology Group-CMI Roullier. Faculty of Sciences. University of Navarra.

Corresponding author:

Andrés Calderín García

e-mail: cg.andres@gmail.com

Tel.: +55 021 3787 3772.

Materials and Methods

Isolation of the humic acids. NaOH (0.1 M) in an atmosphere of N_2 was added to the soil from the treatment with HCl in a ratio of 10:1 (w:v) (in the case of compost and vermicompost the pre-treatment with HCl was not performed and the extraction was performed using the fresh and dried materials) and neutralized to pH = 7 using NaOH (1 M). The mixture was agitated for 8 h and allowed to rest overnight, after which the supernatant was collected via centrifugation (5000 rpm). For the separation by precipitation of the humic acids, an HCl solution (6 M) was added with mixing until pH = 1.0 and allowed to rest for 16 h. Next, the humic acids were separated by centrifugation.

Purification of the humic acids. The humic acids fraction was redissolved in a KOH solution (0.1 M) in an N_2 atmosphere, and a KCl solution (0.3 M K^+) was added to remove suspended solids by centrifugation. The humic acids were reprecipitated by adding HCl (6 M) with mixing until pH = 1.0 and then allowed to rest for 16 h. Next, the supernatant was discarded. The precipitated humic acids were resuspended in an acid mixture (HCl:HF, 0.1:0.1 M) and mixed overnight at room temperature. This procedure was repeated until the ash content was less than 1%. The precipitate was transferred to a dialysis membrane and dialyzed against distilled water until it tested negative for Cl^- ions with $AgNO_3$. The humic acids were later lyophilized.

Isolation and purification of the HS. The HS fraction was obtained from the extraction of the humic fractions of the soil by adding NaOH (0.1 M) in an N_2 atmosphere, at a ratio of 10:1 (w:v) under the same, previously described conditions. The HS extract was later placed on the dialysis membrane (Spectra/Por® 7 dialysis tubing, MWCO 1000) and dialyzed against bidistilled water until the total destaining of the water on the outside of the dialysis tube. Next, the salts were eliminated by passing the HS solution through an ion exchange column (Amberlite IR120 hydrogen form, Fluka analytical Cod.06428). The dissolved HSs were then lyophilized[1,2].

Isolation and purification of soil humins. The humins were obtained using a procedure similar to that described by Nebbioso[3], with some modifications. The solid residue resulting from the extraction of the humic fractions (HS and HA) was washed with distilled water at pH = 7. Next, the resulting solid was lyophilized and then resuspended in a mixture of HF (8%):HCl (4%) v:v with mixing for 16 h in an N_2 atmosphere. The acidic suspension containing the humins was then subjected to dialyses (Spectra/Por® 7 dialysis tubing, MWCO 3500) against distilled water until it tested negative for Cl^- ions and was subsequently lyophilized to obtain the dry and purified humins.

Origin of the composted material. The compost and vermicompost were obtained from the Agroecological Farm of the Embrapa (Fazenda Agroecologica da Embrapa, state of Rio de Janeiro-RJ, Brazil). This is an experimental area that was established in 1993 and is a joint initiative of the Agrobiology Embrapa (Embrapa Agrobiologia), Pesagro-Rio and the Rural Federal University of Rio de Janeiro (Universidade Federal Rural do Rio de Janeiro – UFRRJ). Embrapa developed a technology to produce fertilizers and organic substrates using 100% raw plant materials. These materials are free from biological contamination, do not use mineral fertilizers, and can be certified as organic products[4].

Results and Discussion

Structural characteristics of the humic fractions analyzed by ^{13}C NMR spectroscopy. The peaks present at ~24 ppm and ~32 ppm in the HS and HA fractions correspond to CH_3 ($^*CH_3$-R, R=alkyl) and CH_2 (R-$^*CH_2$-R´, RR´=alkyl) carbons, respectively. In the Hu fractions, the peak representing these structures was recorded at ~29 ppm. The three fractions recorded peaks at ~55 ppm representing CH_3 ($^*CH_3$-O-R, R=alkyl) carbons. The peaks at ~71 ppm represent CH_2 (R-

*CH$_2$OH, R= monosaccharide) carbons, and the peaks at ~102 ppm correspond to anomeric carbons of monosaccharide units. The peaks at ~129 ppm represent aromatic ring carbons (phenyl-R, R=H). Peaks at ~172-175 ppm are attributed to COO (RO-*CO-R´, R=H and R´= aril), and those at ~206-209 ppm are attributed to C=O (R-*CO-R´, RR´=alkyl).

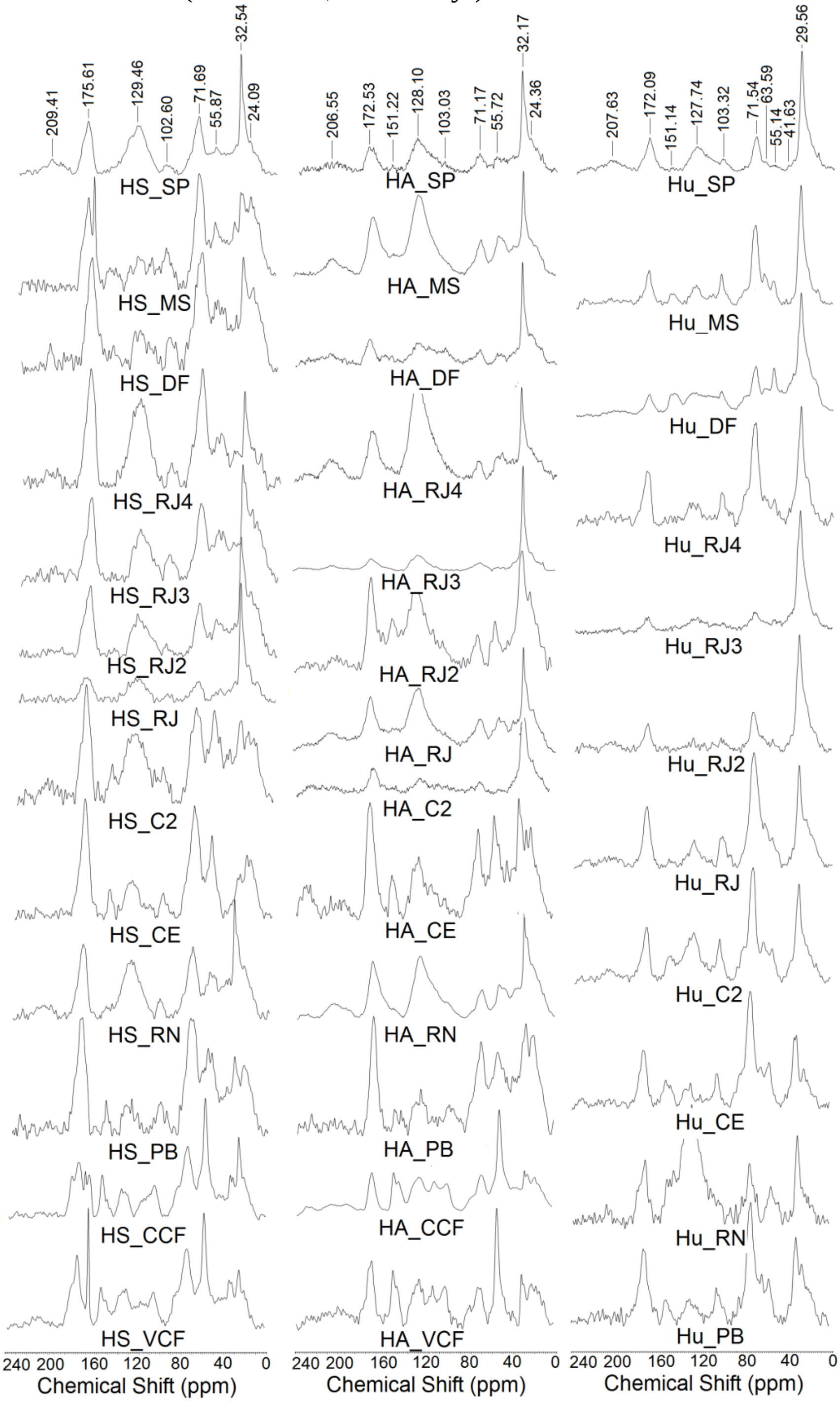

Fig. S1. ^{13}C CP MAS NMR spectra of the different humic fractions extracted from organic soils and composted materials.

Table S1. Relative distributions (percentages) of carbon types in ^{13}C-CP/MAS-NMR spectra.

Humic fraction	CAlk-H,R	CAlk-O,N	C Alk-O	CAlk-di-O	CAr-H,R	CAr-O,N	COO-H,R	C=O	Arom[a]	Aliph[b]
HS_RJ	39.13	6.52	10.87	3.26	17.39	4.35	13.04	5.43	21.74	78.26
HS_RJ2	36.84	11.58	11.58	2.11	15.79	2.11	15.79	4.21	17.89	82.11
HS_RJ3	31.58	12.63	14.74	4.21	15.79	2.11	13.68	5.26	17.89	82.11
HS_RJ4	17.89	11.58	18.95	3.16	21.05	5.26	16.84	5.26	26.32	73.68
HS_DF	28.42	14.74	21.05	4.21	8.42	3.16	14.74	5.26	11.58	88.42
HS_MS	32.98	12.77	17.02	5.32	9,57	4.26	14.89	3.19	13.83	86.17
HS_SP	26.53	10.20	15.31	2.04	23.47	3.06	12.24	7.14	26.53	73.47
HS_PB	24.21	16.84	21.05	4.21	7.37	4.21	17.89	4.21	11.58	88.42
HS_RN	27.37	11.58	15.79	3.16	20.00	3.16	13.68	5.26	23.16	76,84
HS_CE	20.00	13.68	22.11	3.16	11.58	4.21	21.05	4.21	15.79	84.21
HS_C2	23.40	13.83	14.89	4.26	18.09	5.32	13.83	6.38	23,40	76.60
**Mean**	**27.30**	**12.06**	**16.27**	**3.42**	**14.35**	**3.58**	**15.07**	**4.97**	**18.30**	**80.76**
**SD**	**6.35**	**2.53**	**3.60**	**0.95**	**5.15**	**1.06**	**2.43**	**1.05**	**5.26**	**5.26**
HS_VC	18.37	16.33	18.37	6.12	13.27	6.12	17.35	4.08	19.39	80.61
HS_CC	21.43	19.39	18.37	6.12	9.18	5.10	17.35	3.06	14.29	85.71
HA_RJ2	30.68	5.68	5.68	5.68	25.00	6.82	15.91	4.55	31.82	68.18
HA_RJ3	48.89	2.22	7.78	1.11	18.89	1.11	12.22	7.78	20.00	80.00
HA_RJ4	21.43	6.12	3.06	5.10	39.80	3.06	13.27	8.16	42.86	57.14
HA_DF	38.54	4.17	6.25	8.33	18.75	2.08	12.50	9.38	20.83	79.17
HA_MS	22.22	10.10	7.07	5.05	29.29	5.05	15.15	6.06	34.34	65.66
HA_SP	37.89	6.32	7.37	4.21	22.11	2.11	11.58	8.42	24.21	75.79
HA_PB	34.74	12.63	16.84	5.26	8.42	3.16	13.68	5.26	11.58	88.42
HA_RN	31.63	8.16	7.14	5.10	25.51	1.02	15.31	6.12	26.53	73.47
HA_CE	30.00	12.22	14.44	3.33	13.33	4.44	15.56	6.67	17.78	82.22
HA_C2	51.09	1.09	6.52	5.43	10.87	2.17	14.13	8.70	13.04	86.96
HA_RJ	24.24	7.07	9.09	5.05	25.25	5.05	15.15	9.09	30.30	69.70
**Mean**	**32.49**	**5.68**	**7.55**	**4.46**	**19.76**	**2.79**	**13.97**	**7.11**	**23.14**	**74.59**
**SD**	**9.41**	**3.55**	**3.78**	**1.66**	**8.55**	**1.76**	**1.42**	**1.56**	**9.05**	**9.05**
HA_VC	20.00	13.68	9.47	9.47	15.79	9.47	12.63	9.47	25.26	74.74
HA_CC	22.45	18.37	14.29	7.14	18.37	8.16	7.14	4.08	26.53	73.47
Hu_RJ	23.96	5.21	33.33	6.25	9.38	2.08	13.54	6.25	11.46	88.54
Hu_RJ2	46.81	3.19	17.02	5.32	6.38	2.13	12.77	6.38	8.51	91.49
Hu_RJ3	62.11	2.11	10.53	3.16	7.37	1.05	7.37	6.32	8.42	91.58
Hu_RJ4	27.47	5.49	31.87	4.40	6.59	2.20	15.38	6.59	8.79	91.21
Hu_DF	50.52	4.12	12.37	5.15	10.31	5.15	7.22	5.15	15.46	84.54
Hu_MS	30.21	5.21	29.17	7.29	10.42	3.13	10.42	4.17	13.54	86.46
Hu_SP	40.21	2.06	13.40	5.15	16.49	1.03	13.40	8.25	17.53	82.47
Hu_PB	23.08	6.59	27.47	6.59	8.79	4.40	17.58	5.49	13.19	86.81
Hu_RN	15.38	5.49	12.09	4,40	32.97	9.89	9.89	9.89	42.86	57.14
Hu_CE	17.71	8.33	37.50	4.17	6.25	6.25	14.58	5.21	12.50	87.50
Hu_C2	17.89	6.32	34.74	5.26	12.63	4.21	12.63	6.32	16.84	83.16
**Mean**	**29.21**	**4.52**	**21.25**	**5.07**	**10.15**	**3.03**	**11.83**	**6.21**	**13.68**	**84.02**
**SD**	**14.72**	**1.83**	**10.01**	**1.13**	**7.36**	**2.51**	**3.09**	**1.49**	**9.21**	**9.21**

[(a)] **Aromaticity:** Arom = ([CAr-H,R (110-142 ppm) + CAr-O,N (142-156 ppm)/total peak area (0-230 ppm])*100
[(b)] **Aliphaticity:** Aliph = (100 – Arom)

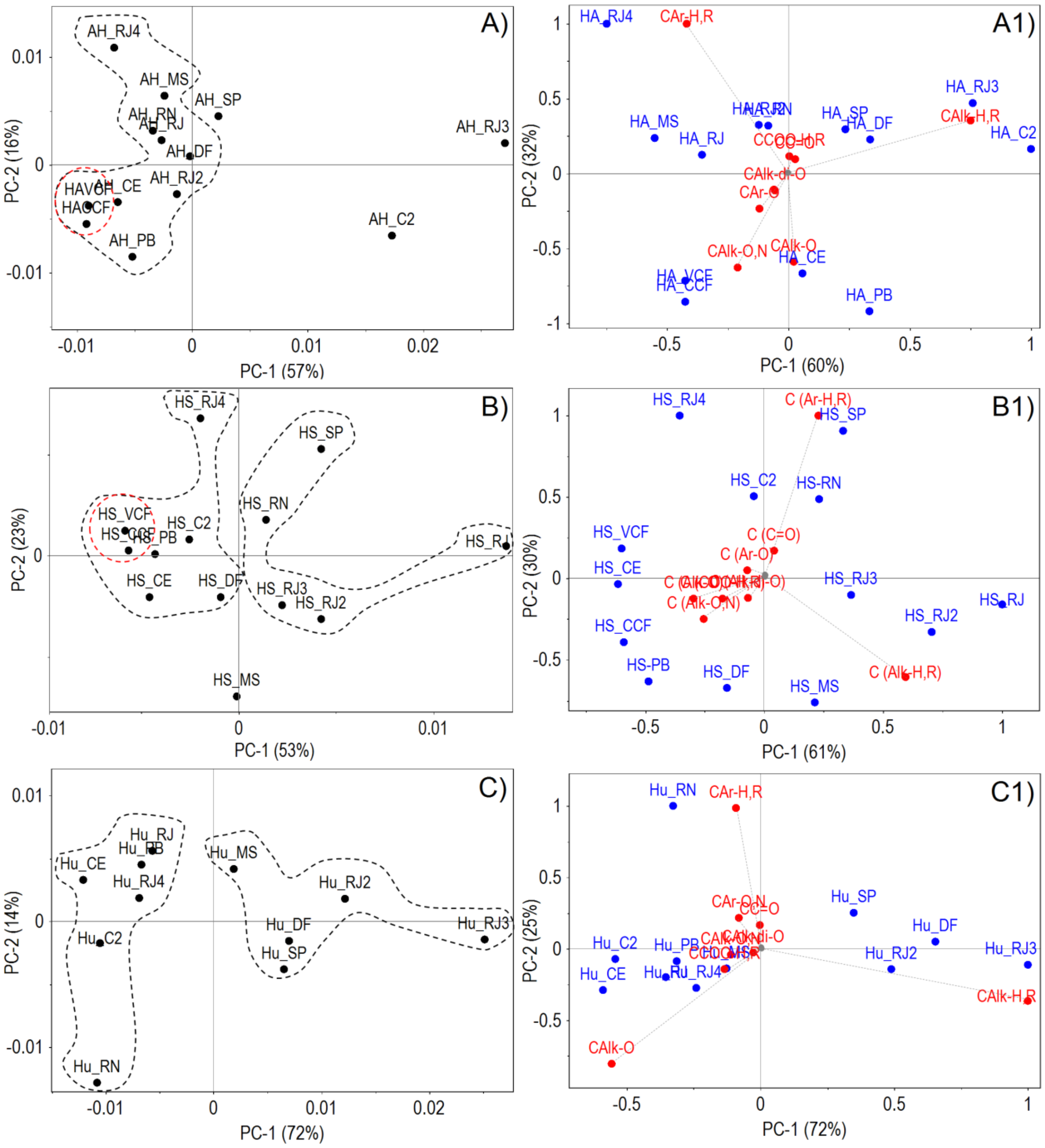

Fig. S2. PCA for the data obtained by loading the ^{13}C-CP/MAS NMR spectra of HSs extracted from soils and composted materials. A, B and C) PCA performed using the pure spectra. A1, B1 and C1) PCA performed through integration by regions in the pure spectra.

Structural characteristics of humic fractions analyzed by FTIR. The absorption bands at ~3400 cm^{-1} indicate the presence of stretching vibrations (ν) –OH and/or –NH (alcohols, carboxylic acids and amides). The bands at ~2900 cm^{-1} and 2800 cm^{-1} correspond to symmetric and asymmetric ν–CH, respectively (–CH_3 aliphatic). An intense band stands out in the HS fractions at ~1593 cm^{-1} corresponding to C=C vibrations of aromatic structures and symmetric and asymmetric ν–COO^-. In the HA fractions, the absorption band at ~1715 cm^{-1} assigned to ν –COOH of carboxylic acids was more visible. Visible bands between 1617 cm^{-1} and 1620 cm^{-1} in the Hu and HA fractions, respectively, are complex bands corresponding to ν C=C aromatic, ν C=O of amide I, and symmetric ν –COO^- (in the HA the asymmetric ν –COO^- was visible at 1407 cm^{-1}). Complex bands at 1388 cm^{-1} and 1378 cm^{-1} visible in HSs and HAs correspond to δ –OH, –CH_2 and –CH_3 deformations and ν –CO of phenols. The bands at ~1035 cm^{-1} and ~1097 cm^{-1} correspond to ν –OH of aliphatic alcohols and polysaccharides.

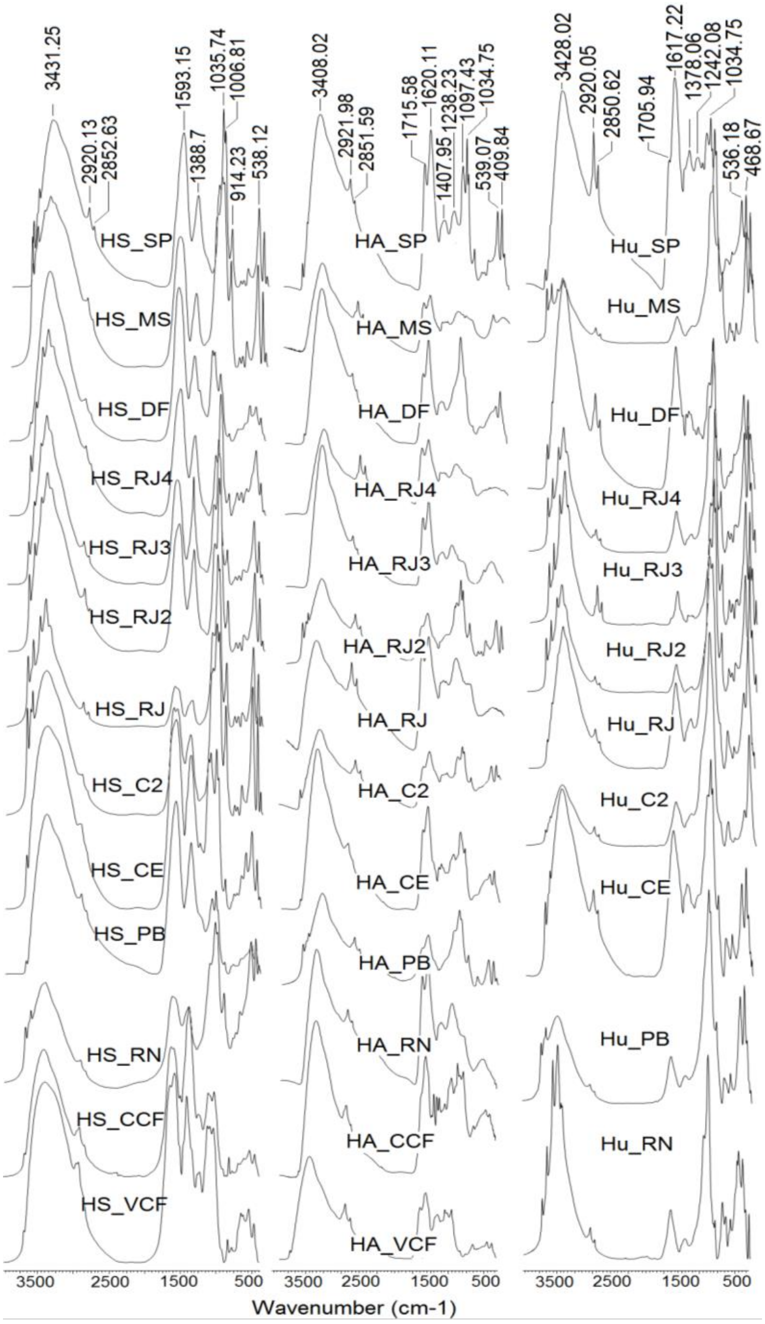

Fig. S3. FTIR spectra of HS extracted from organic soils and composted materials.

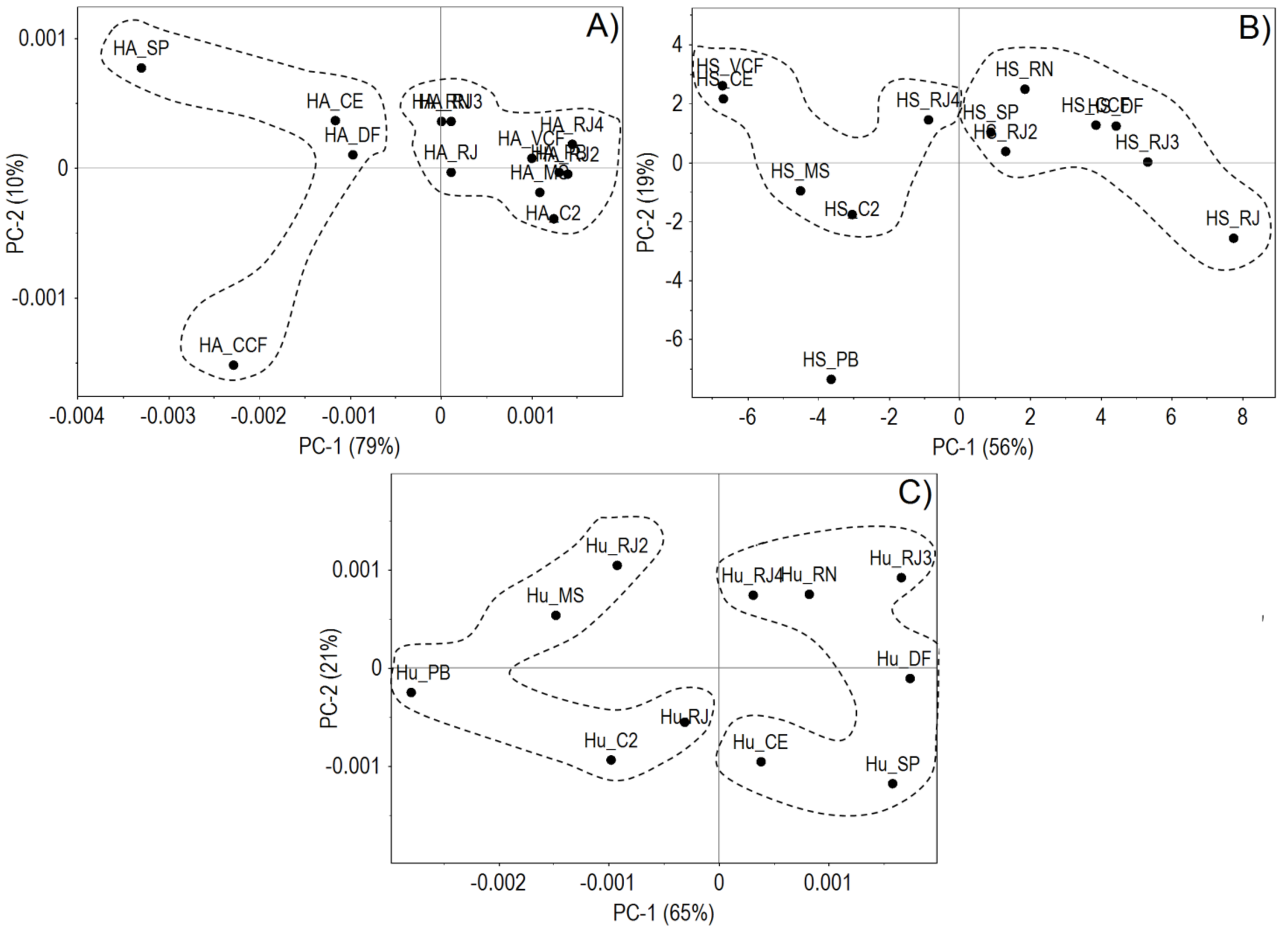

Fig. S4. PCA for the data obtained by loading the FTIR spectra of HSs extracted from organic soils and composted materials.

Table S2. Elemental and isotopic compositions and oxidation parameters, apparent volumes, apparent densities, and E_4/E_6 ratios of HSs.

HS	C	H	N	O	H/C	C/N	O/C	ω	ν	δ	E_4/E_6	$\delta^{13}C$ (‰)
	----------------	---------------- %	----------------	----------------								
HS_RJ	48.17	4.75	2.27	44.35	1.18	24.76	0.69	1.74	0.61	1.64	5.87	-22.687
HS_RJ2	49.26	4.86	3.68	41.72	1.18	15.62	0.64	1.60	0.62	1.61	6.01	-22.153
HS_RJ3	49.07	4.92	4.49	39.58	1.20	12.75	0.60	1.51	0.62	1.62	6.11	-22.192
HS_RJ4	47.26	4.33	3.12	41.81	1.10	17.67	0.66	1.68	0.59	1.70	5.92	-18.623
HS_DF	49.74	4.16	1.40	44.18	1.00	41.45	0.67	1.69	0.60	1.65	5.67	-25.401
HS_SP	47.60	4.52	3.62	42.46	1.14	15.34	0.67	1.69	0.60	1.67	6.21	-17.411
HS_MS	49.19	4.43	3.26	40.50	1.08	17.60	0.62	1.56	0.60	1.65	6.02	-24.937
HS_PB	49.40	3.44	2.52	43.44	0.84	22.87	0.66	1.69	0.58	1.73	5.44	-19.118
HS_RN	49.62	4.14	2.42	43.06	1.00	23.92	0.65	1.65	0.60	1.66	5.77	-25.210
HS_CE	50.23	4.18	3.45	39.60	1.00	16.99	0.59	1.49	0.60	1.65	5.81	-24.552
HS_C2	49.01	4.42	4.37	40.22	1.08	13.08	0.62	1.55	0.60	1.66	6.13	-20.732
HS_CC	53.95	4.05	2.03	40.67	0.90	31.01	0.57	1.43	0.63	1.58	4,35	-14.684
HS_VC	50.06	3.77	4.17	41.72	0.90	14.01	0.63	1.59	0.59	1.68	5,01	-15.005
**Mean**	**49.40**	**4.29**	**2.99**	**41.76**	**1.04**	**19.30**	**0.63**	**1.62**	**0.60**	**1.66**	**5.90**	
**SD**	**1.56**	**0.40**	**0.92**	**1.57**	**0.11**	**7.95**	**0.03**	**0.08**	**0.01**	**0.03**	**0.22**	
HA_RJ	52.40	3.35	3.71	45.34	0.77	16.48	0.65	1.67	0.61	1.65	4.12	-22.483
HA_RJ2	49.49	3.66	2.80	43.03	0.89	20.62	0.65	1.66	0.59	1.70	4.33	-25.124
HA_RJ3	49.68	3.55	2.49	43.64	0.86	23.28	0.66	1.69	0.59	1.71	3.96	-23.595
HA_RJ4	53.10	4.06	2.68	46.36	0.92	23.12	0.65	1.67	0.63	1.58	3.93	-21.948
HA_DF	47.64	4.31	1.98	41.35	1.09	28.07	0.65	1.65	0.59	1.70	4.76	-21.056
HA_MS	52.34	3.12	3.20	46.02	0.72	19.08	0.66	1.70	0.60	1.67	4.66	-25.179
HA_SP	51.40	3.28	2.20	45.92	0.77	27.26	0.67	1.72	0.59	1.68	4.71	-16.604
HA_PB	50.98	3.47	2.26	45.25	0.82	26.32	0.67	1.71	0.60	1.68	4.88	-21.963
HA_RN	51.23	3.44	3.78	44.01	0.81	15.81	0.64	1.65	0.60	1.68	4.49	-24.850
HA_CE	49.92	4.08	4.33	41.51	0.98	13.45	0.62	1.58	0.60	1.66	3.90	-24.101
HA_C2	50.24	3.40	2.60	44.24	0.81	22.54	0.66	1.69	0.59	1.71	4.07	-22.500
HA_CC	52.76	3.04	3.58	46.14	0.69	17.19	0.66	1.69	0.60	1.67	4.23	-14.857
HA_VC	51.25	3.30	3.89	44.06	0.77	15.37	0.64	1.66	0.59	1.69	4.41	-16.108
**Mean**	**50.93**	**3.52**	**2.95**	**44.34**	**0.83**	**20.13**	**0.65**	**1.67**	**0.60**	**1.67**	**4.33**	
**SD**	**1.48**	**0.37**	**0.72**	**1.61**	**0.11**	**4.66**	**0.01**	**0.04**	**0.01**	**0.04**	**0.35**	
Hu_RJ	37.90	3.82	1.91	32.18	1.21	23.21	0.64	1.60	0.48	2.09	---	-18.575
Hu_RJ2	38.36	3.25	1.37	33.74	1.02	32.78	0.66	1.67	0.47	2.14	---	-23.654
Hu_RJ3	31.20	3.38	1.72	26.10	1.30	21.13	0.63	1.56	0.40	2.49	---	-22.787
Hu_RJ4	36.59	3.47	1.24	31.88	1.14	34.30	0.65	1.65	0.46	2.19	---	-26.163
Hu_MS	39.35	3.78	1.47	34.10	1.15	31.32	0.65	1.64	0.49	2.03	---	-26.487
Hu_DF	39.10	4.97	1.54	32.59	1.53	29.70	0.63	1.54	0.53	1.90	---	-26.831
Hu_C2	30.30	3.27	1.46	25.57	1.30	24.29	0.63	1.58	0.39	2.57	---	-22.116
Hu_SP	33.90	4.37	1.36	28.17	1.55	29.02	0.62	1.53	0.46	2.19	---	-27.600
Hu_PB	38.45	3.07	1.57	33.81	0.96	28.63	0.66	1.68	0.46	2.16	---	-20.949
Hu_RN	35.40	3.38	1.83	30.19	1.15	22.59	0.64	1.61	0.44	2.26	---	-25.354
Hu_CE	38.10	4.11	2.11	31.88	1.29	21.05	0.63	1.57	0.49	2.04	---	-23.970
**Mean**	**36.11**	**3.68**	**1.58**	**30.79**	**1.22**	**26.70**	**0.64**	**1.60**	**0.46**	**2.18**	---	
**SD**	**3.02**	**0.55**	**0.25**	**2.90**	**0.18**	**4.58**	**0.01**	**0.05**	**0.04**	**0.19**	---	

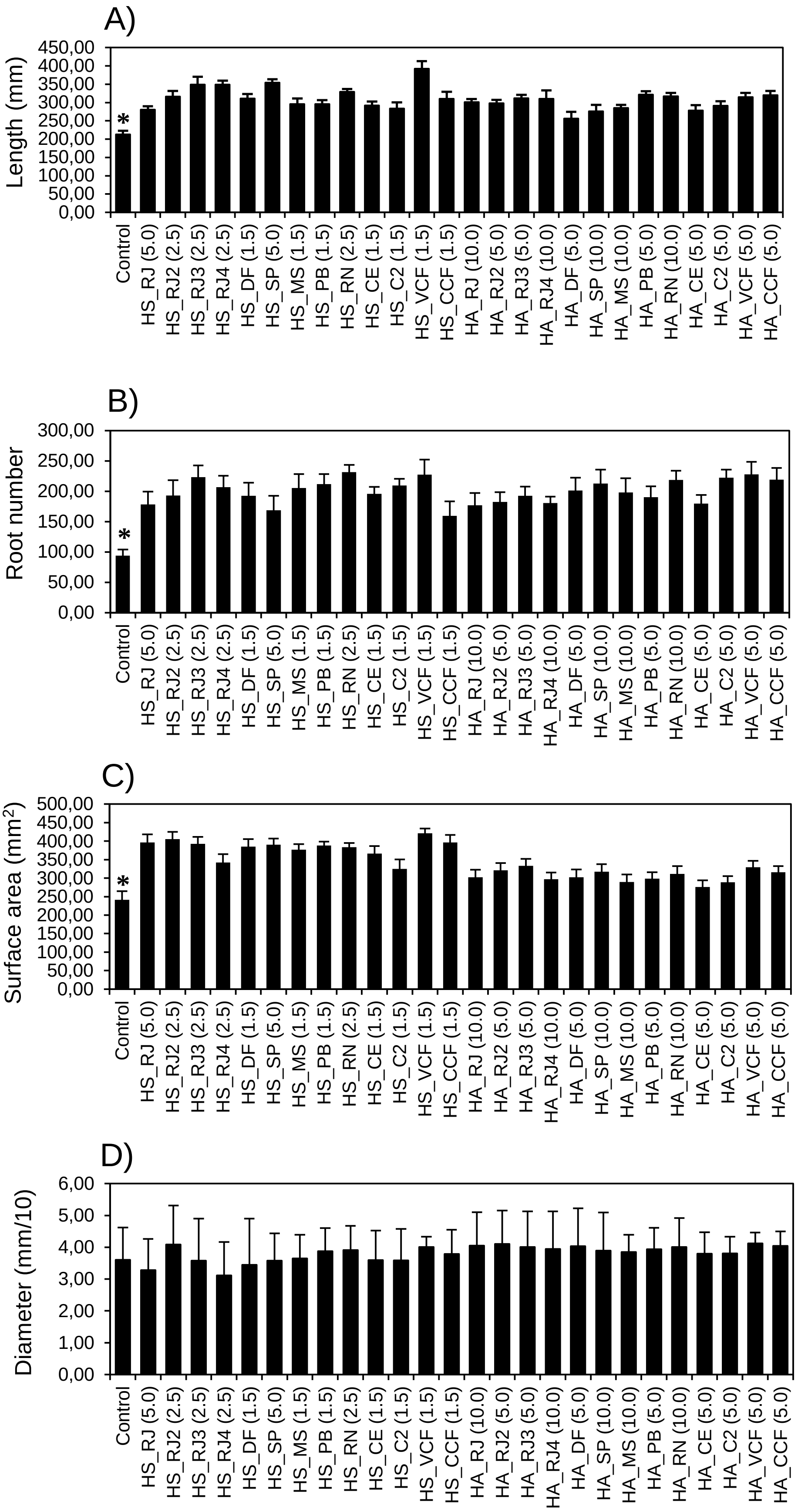

A)
Length (mm)
450,00
400,00
350,00
300,00
250,00
200,00
150,00
100,00
50,00
0,00
*
Control
HS_RJ (5.0)
HS_RJ2 (2.5)
HS_RJ3 (2.5)
HS_RJ4 (2.5)
HS_DF (1.5)
HS_SP (5.0)
HS_MS (1.5)
HS_PB (1.5)
HS_RN (2.5)
HS_CE (1.5)
HS_C2 (1.5)
HS_VCF (1.5)
HS_CCF (1.5)
HA_RJ (10.0)
HA_RJ2 (5.0)
HA_RJ3 (5.0)
HA_RJ4 (10.0)
HA_DF (5.0)
HA_SP (10.0)
HA_MS (10.0)
HA_PB (5.0)
HA_RN (10.0)
HA_CE (5.0)
HA_C2 (5.0)
HA_VCF (5.0)
HA_CCF (5.0)
B)
Root number
300,00
250,00
200,00
150,00
100,00
50,00
0,00
*
C)
Surface area (mm²)
500,00
450,00
400,00
350,00
300,00
250,00
200,00
150,00
100,00
50,00
0,00
*
D)
Diameter (mm/10)
6,00
5,00
4,00
3,00
2,00
1,00
0,00

Continuation...

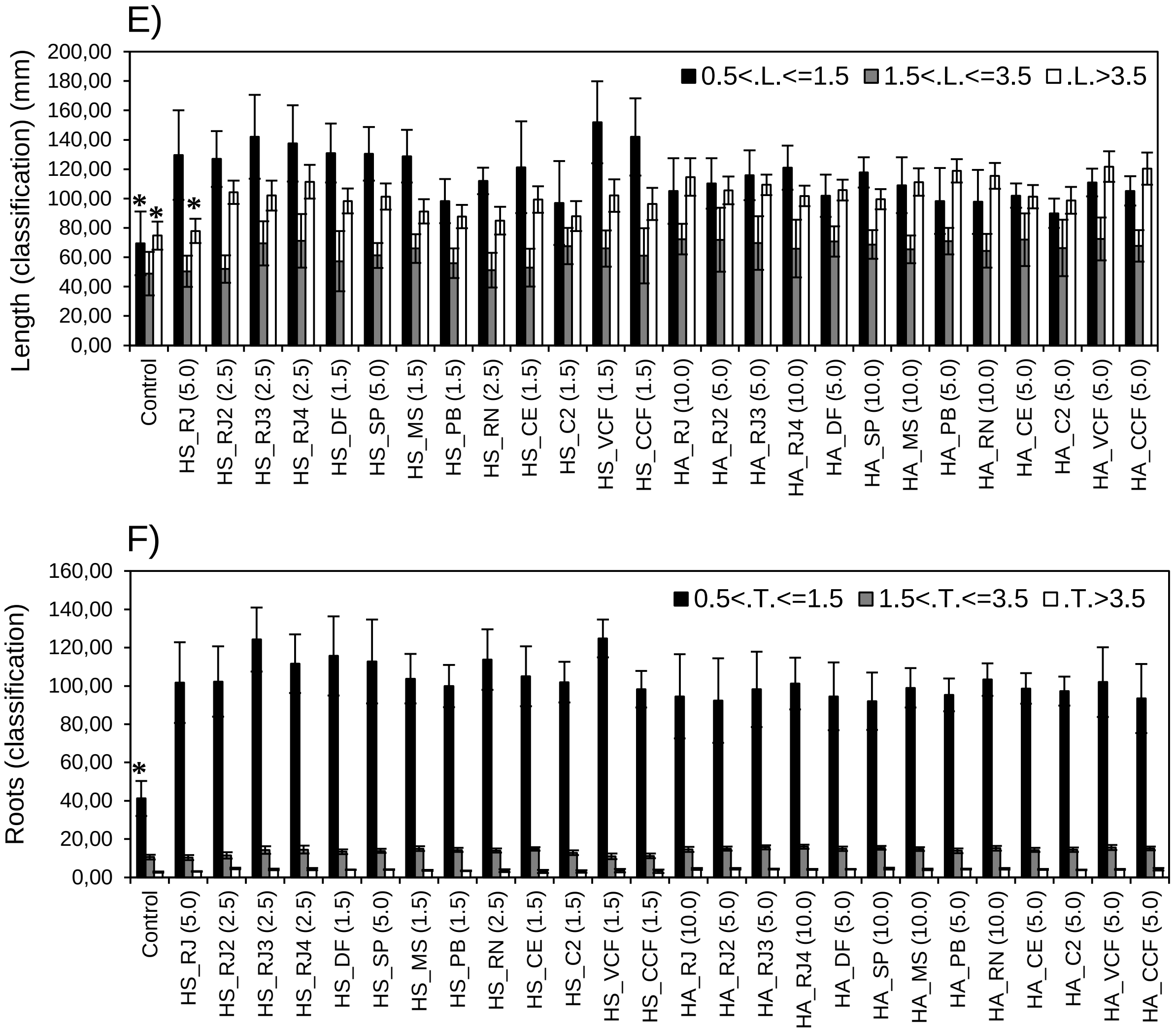

Fig. S5. Bioactivity and root parameters evaluated in rice plants by applying the soluble humic fractions (HSs and HAs) measured using the software WinRhizo. The terms used in each treatment match the nomenclature used for each humic fraction, and the most promising concentrations are indicated in parentheses. A): Main root length, B): Total root number per plant, C): Surface root area per plant, D): Root diameter per plant, E): Root number classification according to the main radicle length, and F): Root number classification according to diameter. Significant differences from the other treatments occur in the plots in which the control treatment is flagged with an asterisk, according to Tukey's test ($p<0.05$), n=12.

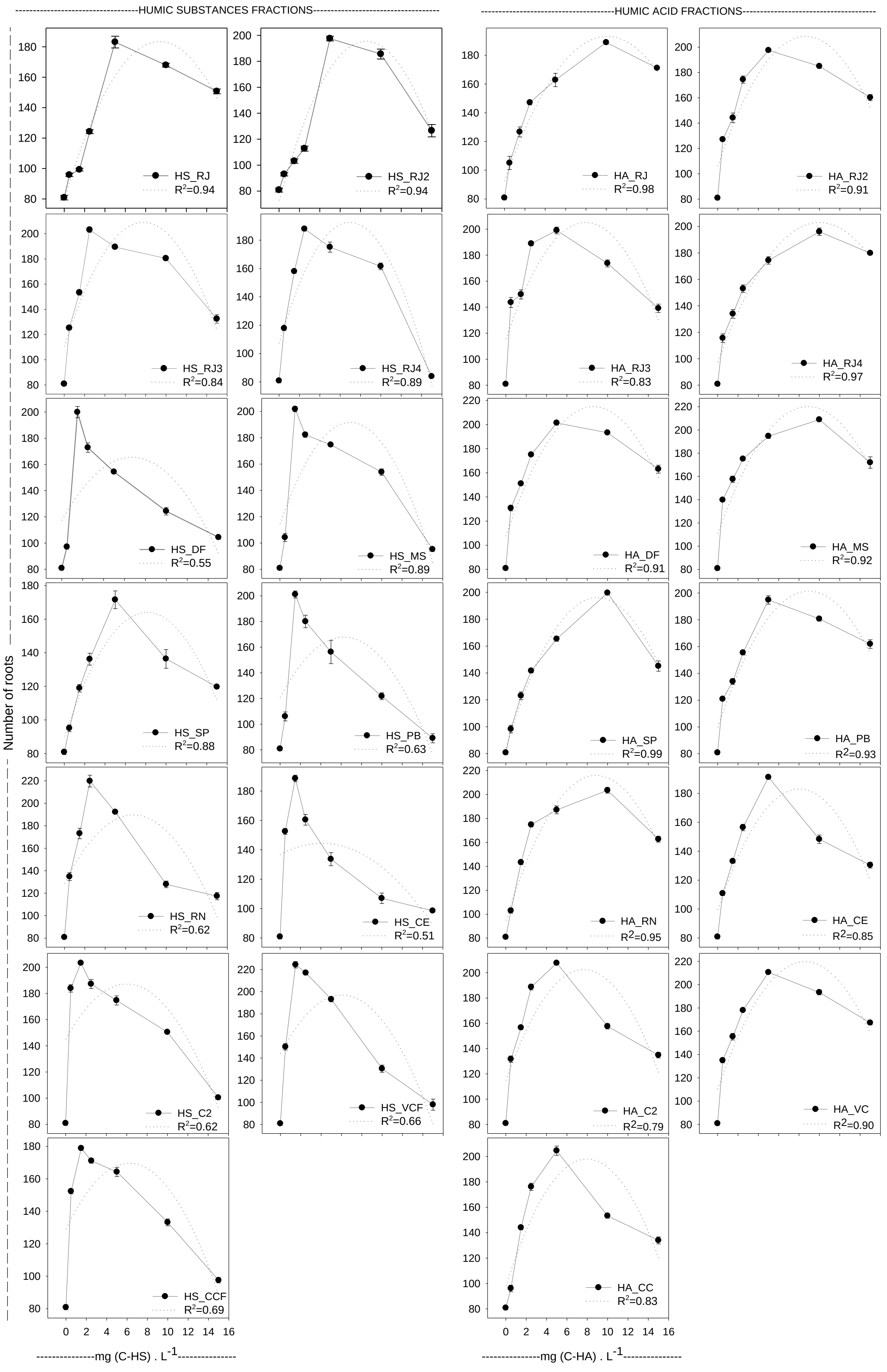

Fig. S6. Root numbers in rice plants in the dose vs. response experiment.

(Additional study) in response to the request from reviewer #1.

In this additional study, the humic fractions of the soils from the State of Rio de Janeiro (humic substances (HS): HS_RJ, humic acids (HA): HA_RJ and fulvic acids (FA): FA_RJ), from the State of Rio Grande do Norte (HS_RN, HA_RN and FA_RN) and from the State of Mato Grosso do Sul, Brazil (HS_MS, HA_MS and FA_MS) were used. Additionally, the humic fractions from the vermicompost (VCF) (HS_VCF, HA_VCF and FA_VCF) were also used.

The FA fractions were extracted according to the procedure described by the IHSS[5]. In general, the liquid humic fraction that remains after centrifugation of the precipitated humic acids was passed through a column loaded with the resin Supelite™ DAX-8, Cod. 21567-U (commercial substitute of Sigma for XAD-8). Next, the fulvic acids retained in the column were eluted with an NaOH solution (0.1 M), and the column was later washed with distilled water for total recovery of the fulvic acids. The fulvic acids were then passed through an ion exchange column (Amberlite IR120 hydrogen form, Fluka analytical Cod. 06428), and the solution was lyophilized.

The structural characterization of the FA was performed as described in the Materials and Methods section of the manuscript.

The bioactivity experiments were carried out under the same conditions as those described in the Materials and Methods section of the manuscript.

The root parameters were evaluated as described in the Materials and Methods section of the manuscript.

The structure-activity relationship was established as described in the Materials and Methods section of the manuscript.

Results and Discussion

^{13}C NMR and PCA characterization of the spectral data from the different humic fractions.

As expected, the soil-extracted FAs were shown to have structural characteristics independent of the HA and HS fractions. However, the data confirm our research hypothesis and reaffirm the results reported in the manuscript because the HA and HS fractions of the VCF are structurally similar to the whole HS fractions extracted from the soils. In the PCA-^{13}C NMR (71% of the total variance explained), the negative values on PC1 (55%) indicate that the FA extracted from the VC are structurally similar to the whole HS fractions (Fig. S7). This same behavior was observed when comparing the soil HA and HS fractions between the compost and vermicompost using PCA (Figure 1-manuscript).

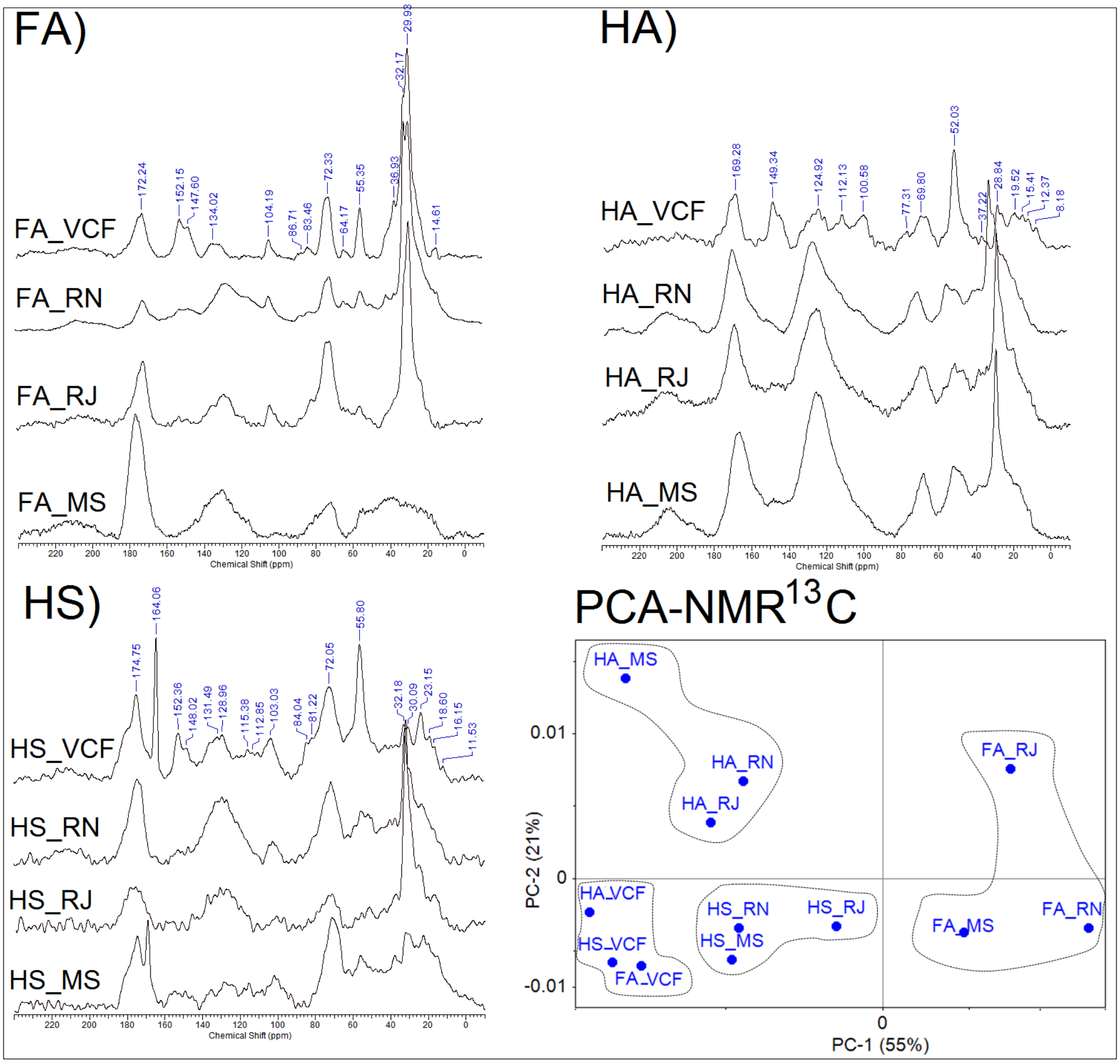

Fig. S7. ^{13}C CP MAS NMR spectra and principal component analyses (PCA) of the fractions extracted from the soils and vermicompost. FA: fulvic acids, HA: humic acids and HS: whole humic substances.

Biological Activity

The FA fractions were shown to have biological activity, stimulating plant root growth; these results have already been observed in the literature[6,7]. The FA concentrations (2.5-5.0 mg (C) .L^{-1}) with the greatest stimulation of root growth were lower than those for the HAs (5.0-10.0 mg (C) .L^{-1}). Thus, the FAs showed a range of maximum effect similar to that shown by the HS (Fig. S8).

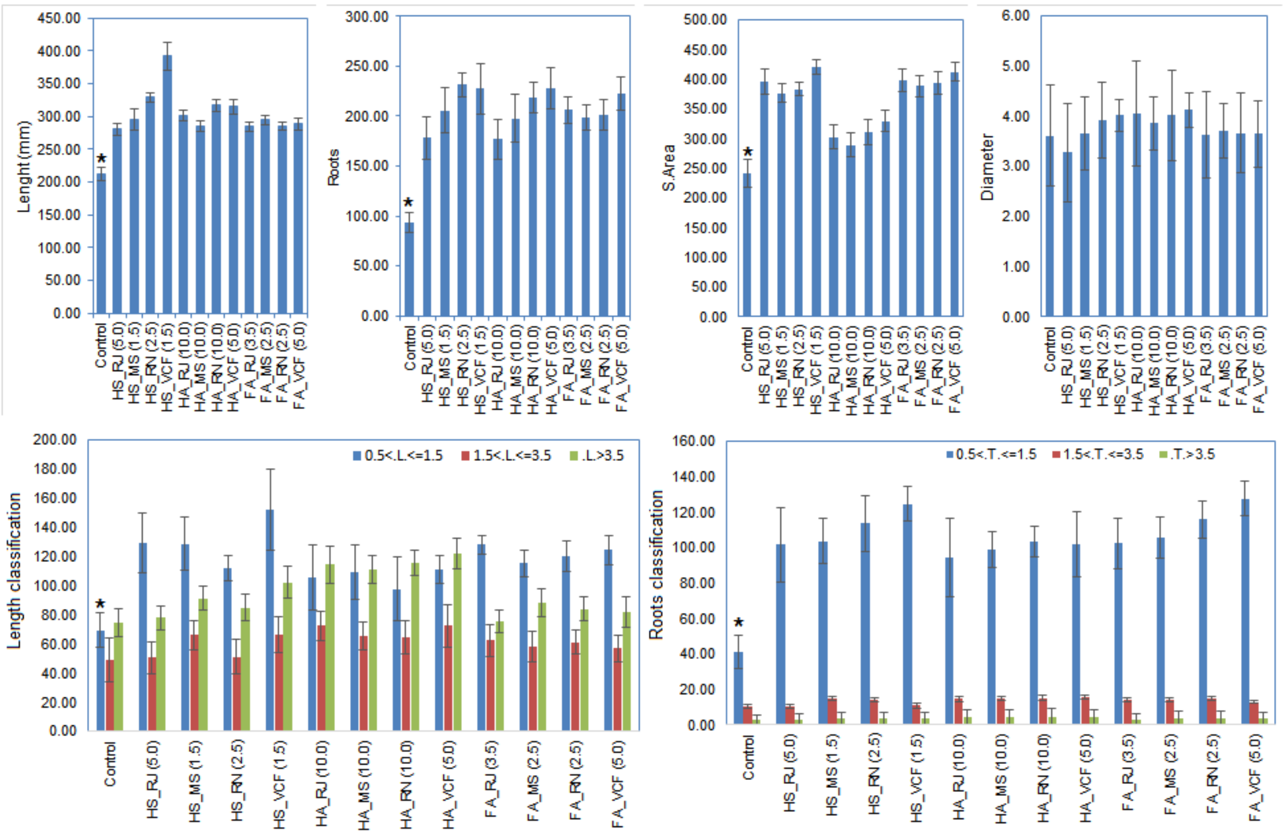

Fig. S8. Bioactivity and root parameters evaluated in rice plants by applying the soluble humic fractions (HSs, Has and FA) measured using the software WinRhizo. The terms used in each treatment match the nomenclature used for each humic fraction, and the most promising concentrations are indicated in parentheses. Significant differences from the other treatments occur in the plots in which the control treatment is flagged with an asterisk, according to Tukey's test ($p<0.05$), n=12.

The PCA (90% of the total variance explained) that relates the humic fractions to the root parameters is shown in Fig. S9A. The plot shows that both the FA fractions as well as the whole HSs (positive values on PC1—80%) exert effects on the smaller roots and on the number of smaller roots. These results can be interpreted as effects on the emission as well as the length and surface area of the roots. The HAs were shown to exert effects on the diameter of the roots and larger roots, which can be interpreted as effects that are more related to root growth and vigor. The PCA (90% of the total variance explained) that relates these effects with the structures is shown in Fig. S9B. The HS and FA fractions showed a relationship (positive values on PC1—80%) between predominantly more functionalized structures (C-substituted) and their effects on root emission, in contrast to the HAs, which showed a relationship with negative values on PC1 for structures that were predominantly less functionalized (C-unsubstituted) and root growth and diameter parameters. These results again confirm the findings of the studies shown in the manuscript and prove that the most functionalized structures could be responsible for the emission of roots within the mode of action of the HSs on plants, while the less functionalized structures could exert more direct effects on root growth and thickening (Fig. S9).

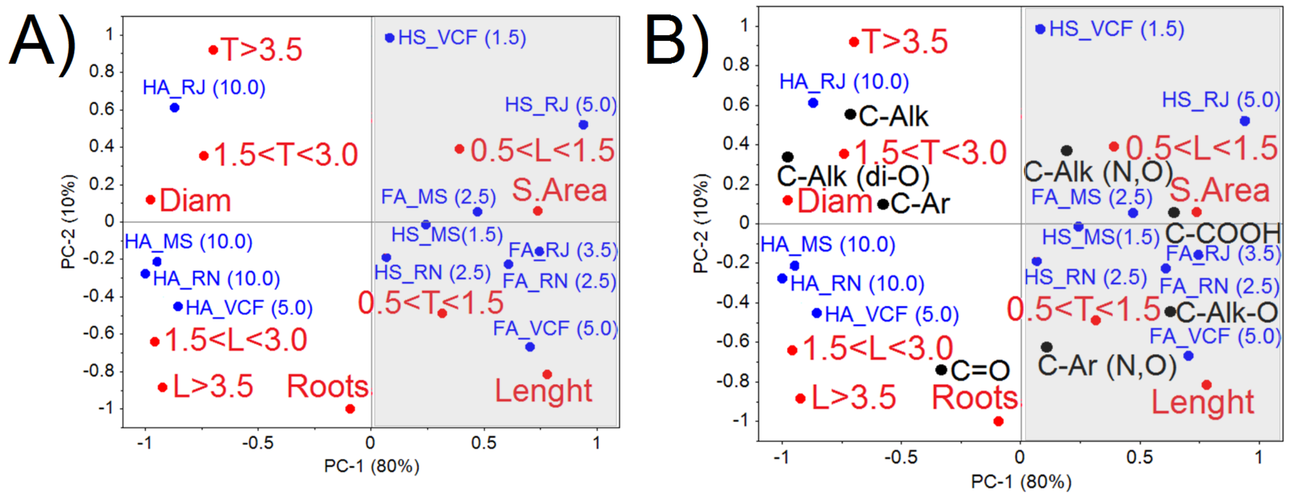

Fig. S9. PCA showing the relationship between the data resulting from the quantification of carbon types in the ^{13}C-CP/MAS-NMR spectra (A) and from the elemental analysis (B) of humic fractions (HSs and HAs) with the root parameters evaluated in rice plants.

Fig. S10 shows the MCR, which enables determination of the patterns of recalcitrance and lability of the FA fractions. As seen in the manuscript and as demonstrated for the HA, HS and Hu fractions, the technique enables obtaining a spectral pattern of recalcitrance from non-functionalized carbon structures (C-alk (H,R) and C-arm (H,R) and associated COOH groups, while the pattern of lability originates from the predominantly functionalized structures (C-alk (O,N), C-alk-O, C-alk di-O, C-arm (O,N)) and COOH.

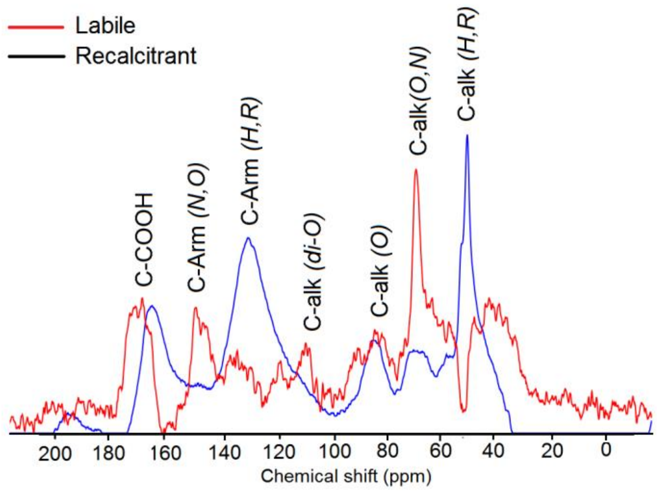

Fig. S10. Multivariate curve resolution (MCR) performed by loading the ^{13}C-CP/MAS-NMR spectra of FA from organic soils.

Fig. S11 shows the PCR performed to relate the structural pattern of the FAs to the root parameters. The FAs showed a positive correlation of the labile structures with the effects exerted on the plant roots. This pattern of positive correlation is similar to the lability pattern shown and obtained through the MCR analysis and is also similar to that shown by the HS fractions (Figure 6). These

results confirm our research hypothesis and prove the conclusions obtained based on the results of the manuscript.

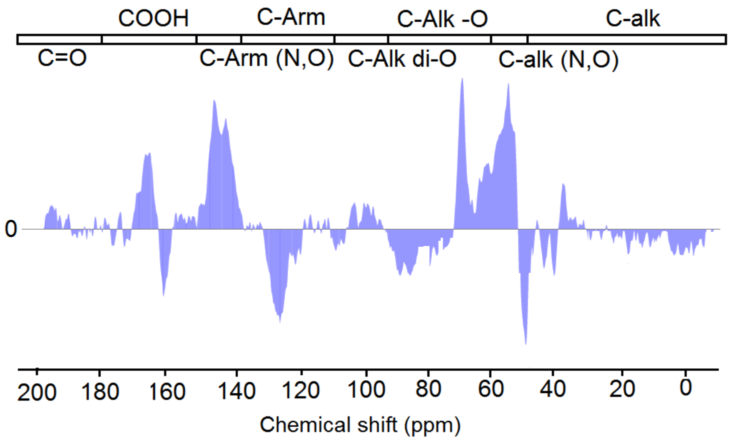

Fig. S11. Principal component regression (PCR) of data from the ^{13}C-CP/MAS-NMR spectra and the root parameters of plant bioactivity.

Conclusions

The results presented in these new experiments are in agreement with the conclusions of the main manuscript. The lability, a property resulting from the structural characteristics and obtained through a statistical tool, is shown again as a viable parameter for the interpretation of the relationships between the structure, property and functions of the HSs, and which was also confirmed in this case for the FAs. Additionally, this experiment suggests the existence of a supramolecular structural organization of the HSs as a whole. Our results indicate that the hydrophobic and hydrophilic domains of the HSs show a type of specific action in the regulation of root growth. Our results are coherent with those published by Canellas[8], where it was proven that the hydrophobic domain of the HSs can be broken to release the hydrophilic structures responsible for the stimulation and emission of roots in plants.

Table S3. Chemical properties of the Histosols used in this study. The mean values for 3 replicates.

**Histosoil (*)**	**pH (water)**	**pH (KCl)**	**Ca^{2+}**	**Mg^{2+}**	**K^{+}**	**Na^{+}**	**Al^{3+}**	**H^{+}**	**H+Al**	**CEC**
**RJ**	4.84±0.05	4.32±0.12	0.50±0.12	1.13±0.03	0.27±0.014	0.36±0.01	0.90±0.012	14.28±1.21	15.18±0.324	17.50±1.25
**RJ2**	3.95±0.32	3.50±0.17	1.00±0.15	1.53±0.12	0.21±0.011	0.67±0.02	3.80±0.034	28.21±1.54	32.01±0.854	35.40±1.52
**RJ3**	5.04±0.11	4.40±0.36	6.60±0.24	3.90±0.21	0.34±0.012	0.97±0.02	0.87±0.058	16.29±1.32	17.16±0.924	29.00±2.13
**RJ4**	5.50±0.54	4.30±0.45	2.50±0.31	4.70±0.15	0.12±0.051	0.26±0.03	0.10±0.01	20.20±1.62	20.30±0.963	27.90±3.01
**DF**	5.90±0.18	4.70±0.85	8.60±1.02	10.2±0.72	0.27±0.034	2.02±0.01	0.00	12.21±1.27	12.20±0.945	33.30±3.11
**SP**	5.20±0.44	3.20±0.71	0.60±0.02	1.50±0.01	0.45±0.028	0.61±0.04	0.40±0.018	57.85±1.98	58.20±0.932	61.40±2.14
**MS**	7.50±0.27	4.50±0.84	42.70±2.01	4.30±0.13	0.39±0.034	0.24±0.01	0.00	1.60±0.12	1.60±0.002	49.20±1.65
**PB**	6.00±0.09	5.60±0.09	53.80±1.45	4.40±0.19	0.13±0.039	1.55±0.05	0.00	4.60±0.14	4.60±0.023	64.50±1.84
**RN**	7.60±0.71	7.10±0.75	47.20±1.98	15.30±0.97	0.24±0.022	1.41±0.02	0.00	0.20±0.01	0.20±0.001	64.40±2.51
**CE**	6.10±0.62	5.70±0.43	24.00±1.11	25.10±1.02	0.70±0.047	9.46±0.95	0.20±0.017	6.20±0.12	6.40±0.001	65.60±3.22
**C2**	5.20±0.50	4.60±0.55	10.80±1.06	2.90±0.21	0.04±0.001	0.48±0.03	0.10±0.02	6.20±0.25	6.30±0.234	20.60±3.41

**Histosoil (*)**	**S**	**V**	**P**	**OM**	**C**	**N**	**C/N**	**C-HA**	**C-FA**	**C-Hu**	**AH/AF**
**RJ**	2.30±0.12	13.00±2.15	37.00±2.14	148.8±21.4	66.20±11.5	7.40±1.02	9.00±1.03	15.80±0.21	16.17±0.55	31.46±1.03	1.02±0.02
**RJ2**	3.40±0.24	10.00±1.24	3.00±0.35	745.8±10.4	368.0±10.3	16.30±1.1	23.00±1.21	88.10±0.35	10.80±0.84	253.82±9	11.61±1.02
**RJ3**	11.80±1.02	41.00±1.47	38.00±1.55	222.6±44.5	116.0±14.3	8.50±0.54	14.00±2.03	35.75±0.85	16.22±0.95	60.11±7.11	2.33±0.03
**RJ4**	7.60±0.13	27.00±1.18	23.00±1.24	186.0±31.5	168.3±10.6	5.20±0.98	18.00±1.55	30.50±0.42	15.60±0.92	20.90±3.21	1.95±0.056
**DF**	21.00±1.21	63.00±1.16	25.00±1.31	187.0±33.8	145.8±9.54	5.80±0.58	18.00±1.63	37.80±0.62	11.80±0.89	38.60±3.22	3.21±0.04
**SP**	3.20±0.34	5.00±1.14	4.00±0.15	391.0±27.9	231.4±21.3	9.40±0.32	22.00±1.31	112.80±0.34	23.70±0.99	53.70±2.45	4.76±0.24
**MS**	47.60±2.15	97.00±2.31	77.00±1.84	254.0±26.4	95.40±17.1	6.89±0.74	13.78±2.3	1.40±0.55	2.80±0.12	62.80±9.87	0.50±0.11
**PB**	59.90±2.35	93.00±2.47	17.00±1.47	211.0±20.1	85.41±10.9	7.86±0.24	10.05±2.14	10.58±0.84	4.43±0.11	74.70±4.56	2.39±0.36
**RN**	64.20±2.54	100.0±3.55	15.00±1.63	174.5±20.6	69.81±11.8	4.81±0.65	15.83±2.51	2.94±0.27	2.17±0.2	71.17±8.36	1.19±0.24
**CE**	59.20±3.65	90.00±3.64	8.00±1.42	504.0±19.5	257.6±23.6	17.1±0.88	14.39±2.22	36.00±0.51	14.82±2.01	212.82±10.1	2.43±0.17
**C2**	14.30±2.44	69.00±3.19	69.00±3.94	118.0±16.9	42.52±11.9	4.13±0.11	12.01±2.33	11.89±0.56	5.24±1.03	38.75±6.66	2.27±0.61

pH H_2O (1:2.5)
pH KCl (1:2.5)
Ca^{2+}: (cmolc.kg^{-1})
Mg^{2+}: (cmolc.kg^{-1})
K^{+}: (cmolc.kg^{-1})
Na^{+}: (cmolc.kg^{-1})
Al^{3+}: (cmolc.kg^{-1})
H^{+}: (cmolc.kg^{-1})
H+Al: (cmolc.kg^{-1})
CEC: (cmolc.kg^{-1})
S: (cmolc.kg^{-1})
V: (%)
P: (mg.kg^{-1}) (Available phosphorus)
OM: (g.kg^{-1}) (Organic matter)
TOC: (g.kg^{-1}) (Total organic carbon)
N: (g.kg^{-1})
C-AH: (g.kg^{-1}) (carbon as humic acid)
C-FA: (g.kg^{-1}) (carbon as fulvic acid)
C-Hu (g.kg^{-1}) (carbon as humin)
(*): As organic carbon is prevalent in these soils, particle size studies are not commonly performed

Table S4. Properties of vermicompost and compost materials. The mean values for 4 replicates.

	**Vermicompost**	**Compost**
pH	6.46±0.14	6.8±0.14
C.E (µS .cm^{-1})	254.00±10.58	196.00±8.12
N (g.kg^{-1})	5.70±1.03	4.80±0.98
Ca^{2+} (g.kg^{-1})	2.02±0.21	11.40±1.02
Mg^{2+} (g.kg^{-1})	4.45±0.69	8.30±0.89
P (g.kg^{-1})	1.51±0.01	5.12±1.03
K^{+} (g.kg^{-1})	5.00±1.03	14.00±1.84
COT (g.kg^{-1})	62.44±2.87	57.47±7.22
C:N	10.95±0.87	11.97±1.54
HA (g.kg^{-1})	15.71±0.64	12.10±1.62
FA (g.kg^{-1})	9.90±0.77	11.42±1.11
HA/FA	1.58±0.03	1.05±0.02

References

1. Pizzeghello, D., Nicolini, G., & Nardi, S. Hormone-like activity of humic substances in Fagus sylvaticae forests. *New Phytol.* **151,** 647-657 (2001). doi: 10.1046/j.0028-646x.2001.00223.x.
2. Dell'Agnola, G., & Ferrari, G. Molecular sizes and functional groups of humic substances extracted by 0.1 m pyrophosphate from soil aggregates of different stability. *J Soil Sci*. **22**, 342-349 (1971). doi: 10.1111/j.1365-2389.1971.tb01622.x
3. Nebbioso, A., Vinci, G., Drosos, M., Spaccini, R., & Piccolo, A. Unveiling the molecular composition of the unextractable soil organic fraction (humin) by humeomics. *Biol. Fert. Soils*. **51**, 443-451. (2015).
4. Empresa Brasileira de Pesquisa Agropecuária (EMBRAPA). *Embrapa agrobiologia*. Available at: https://www.embrapa.br/agrobiologia. (Accessed: 31 October 2015).
5. Swift, R. S. *Method for extraction of IHSS soil fulvic and humic acids* (eds de Sparks, Page A, Helmke, PA, Loeppert, RH) 1018-1020 (1996).
6. Canellas, L. P., *et al*. Humic and fulvic acids as biostimulants in horticulture. *Scientia Horticulturae* (2015). doi:10.1016/j.scienta.2015.09.013
7. Lotfi, R., Pessarakli, M., Gharavi-Kouchebagh, P., & Khoshvaghti, H. Physiological responses of Brassica napus to fulvic acid under water stress: chlorophyll a fluorescence and antioxidant enzyme activity. *Crop J*. **3**, 434-439. (2015). doi:10.1016/j.cj.2015.05.006
8. Aguiar, N. O., *et al*. Prediction of humic acids bioactivity using spectroscopy and multivariate analysis. *J. Geochem. Explor*. **129**, 95-102. (2013). doi:10.1016/j.gexplo.2012.10.005
